# CCL5/RANTES contributes to hypothalamic insulin signaling for systemic insulin responsiveness through CCR5

**DOI:** 10.1038/srep37659

**Published:** 2016-11-29

**Authors:** Szu-Yi Chou, Reni Ajoy, Chun Austin Changou, Ya-Ting Hsieh, Yang-Kao Wang, Barry Hoffer

**Affiliations:** 1 The PhD Program for Neural Regenerative Medicine, College of Medicine, College of Medical Science and Technology, Taipei Medical University and National Health Research Institutes (NHRI), 250 Wuxing St. Taipei, Taiwan, 110; 2 Graduate Institute of Neural Regenerative Medicine, College of Medical Science and Technology, Taipei Medical University, Taipei, Taiwan; 3 Center for Neurotrauma and Neuroregeneration, College of Medical Science and Technology, Taipei Medical University, Taipei, Taiwan; 4 The Ph.D. Program for Cancer Biology and Drug Discovery, College of Medical Science and Technology, Taipei Medical University, Taipei, Taiwan; 5 Integrated Laboratory, Center of Translational Medicine, Taipei Medical University, Taipei, Taiwan; 6 Core Facility, Taipei Medical University, Taipei, Taiwan; 7 Department of Cell Biology and Anatomy, College of Medicine, National Cheng Kung University, Tainan, Taiwan; 8 Department of Neurosurgery, Case Western Reserve University School of Medicine, Cleveland, OH, USA

## Abstract

Many neurodegenerative diseases are accompanied by metabolic disorders. CCL5/RANTES, and its receptor CCR5 are known to contribute to neuronal function as well as to metabolic disorders such as type 2 diabetes mellitus, obesity, atherosclerosis and metabolic changes after HIV infection. Herein, we found that the lack of CCR5 or CCL5 in mice impaired regulation of energy metabolism in hypothalamus. Immunostaining and co-immunoprecipitation revealed the specific expression of CCR5, associated with insulin receptors, in the hypothalamic arcuate nucleus (ARC). Both *ex vivo* stimulation and *in vitro* tissue culture studies demonstrated that the activation of insulin, and PI3K-Akt pathways were impaired in CCR5 and CCL5 deficient hypothalamus. The inhibitory phosphorylation of insulin response substrate-1 at Ser302 (IRS-1^S302^) but not IRS-2, by insulin was markedly increased in CCR5 and CCL5 deficient animals. Elevating CCR5/CCL5 activity induced GLUT4 membrane translocation and reduced phospho-IRS-1^S302^ through AMPKα-S6 Kinase. Blocking CCR5 using the antagonist, ^Met^CCL5, abolished the de-phosphorylation of IRS-1^S302^ and insulin signal activation. In addition, intracerebroventricular delivery of ^Met^CCL5 interrupted hypothalamic insulin signaling and elicited peripheral insulin responsiveness and glucose intolerance. Taken together, our data suggest that CCR5 regulates insulin signaling in hypothalamus which contributes to systemic insulin sensitivity and glucose metabolism.

CCL5/RANTES (C-C motif ligand 5, Regulated-on-Activation-Normal-T-cell-Expressed-and-Secreted) belongs to the C-C chemokine group with multiple functions in the organism. We have previously demonstrated that CCL5/RANTES is an important neurotrophic factor which promotes cortical neurite outgrowth and cortical neuron activity in a Huntington’s disease animal model^1^ and which is also a downstream factor for promoting axonal genesis by hepatocyte growth factor^2^. Many neurodegenerative diseases such as Parkinson’s disease (PD), Huntington’s disease (HD) and Alzheimer’s disease (AD) manifest mitochondrial dysfunction and energy metabolism impairment, insulin resistance and Type 2 diabetes mellitus (T2DM)^3^,^4^,^5^,^6^. Huntington’s disease, for example, shows severe abnormalities in insulin sensitivity, impaired glucose homeostasis, and elevated 5′ AMP-activated protein kinase (AMPKα) activity in both patients and animal disease models^7^,^8^,^9^. The impaired energy metabolism and insulin resistance accelerates neurons degeneration. Interestingly, there are many studies indicating that CCL5/RANTES and its receptor–CCR5 are also associated within T2DM, glucose intolerance, obesity, atherosclerosis and HIV infection-induced metabolic changes^10^,^11^,^12^,^13^,^14^,^15^,^16^. However, the roles and mechanisms of CCL5/RANTES and CCR5 actions in insulin function and glucose metabolism remain under debate. Kitade and co-workers reported that CCR5 deficiency protected mice from obesity-induced inflammation, macrophage recruitment and insulin resistance^17^. Kennedy and colleagues, in contrast, reported that CCR5 deficiency impairs systemic glucose tolerance as well as adipocyte and muscle insulin signaling^18^. Both studies used high fat (HF) diets to generate long-term excess energy uptake and to induce insulin insensitivity and glucose intolerance in mice with very different results; the fundamental function and mechanisms of CCL5-CCR5 effects in insulin and glucose metabolism are thus important to delineate with further experiments.

Neurodegenerative diseases (NDD) and T2DM also share one additional common mechanism-chronic inflammation. Inflammatory cytokines and chemokines cause neuron death and degeneration in NDD, and pancreatic islet and liver dysfunction in T2DM. Many cytokines have specific functions in the hypothalamus rather than only inflammation. TNF-α in the hypothalamus inhibits food intake, whereas mice without TNF-α receptors develop obesity and diabetes^19^,^20^. IL-6 contributes to the expression of hypothalamic neuropeptides for body weight regulation^21^. CCL5/RANTES also functions as a neuroendocrine element regulating food intake and body temperature in the hypothalamus via unidentified receptors^22^,^23^,^24^. One of CCL5/RANTES’s receptors, GPR75 (G-protein receptor 75), on the pancreatic β-cell membrane is involved with stimulating insulin secretion and improves glucose homeostasis in both lean mice and *ob/ob* insulin resistant mice^25^. Recent studies have shown the existence of a central nervous lymphatic system which increases the importance of chemokines transport from periphery to the central nervous system^26^,^27^. Therefore, the role of CCL5/RANTES and its receptors in central hypothalamus function is important to be investigated.

The hypothalamus plays a central role in the regulation of body energy homeostasis and fuel sensing in the brain. The melanocortin system within the ARC coordinates cellular energy status and hormones such as insulin and adipokines-leptin from the periphery to regulate systemic glucose flux, insulin responsiveness, food intake, and energy expenditure. The insulin receptor substrates (IRS), binding with the insulin receptor (IR), function as key regulators in insulin signaling. IRSs can positively or negatively regulate the insulin signal through phosphorylating different tyrosine or serine residues^28^,^29^. Impairment of IR or IRS proteins activity in the hypothalamus directly leads insulin resistance and glucose intolerance in mice^30^,^31^,^32^,^33^,^34^. AMPKα also participates in the hypothalamic pro-opiomelanocortin (POMC) and Agouti-related peptide (AgRP) neuron regulation of food intake as well as body weight maintenance by integrating signals from circulating levels of insulin, leptin and glucose^35^,^36^,^37^. A rise in AMPK levels in hypothalamus increases food intake^35^ which is then reduced after feeding^38^. Elevated AMPK also increases hepatic glucose production, muscle glycogen synthesis^39^ and glucose uptake via enhancing GLUT4 membrane translocation^40^,^41^. Mice lacking AMPKα2 show no response to glucose^42^. Interestingly, CCL5 has been found to regulate glucose uptake and AMPK signaling in T-cells^43^. It is thus important to investigate CCL5 in glucose uptake and AMPK signaling for hypothalamic neuron function.

The present study focuses on investigation of the role and potential mechanisms of CCL5/RANTES and CCR5 signaling in energy metabolism regulation, glucose intolerance, and insulin resistance in the hypothalamus. We demonstrate here that mice lacking CCL5 or CCR5 have impaired hypothalamic neurons energy regulation with reduced insulin signal activation both *ex vivo* and *in vitro*. CCR5 associates with the insulin receptor and influences pathways for GLUT4 membrane translocation. Such mechanisms play a “checkpoint” role for insulin signal negative feedback regulation. Blocking CCR5 in hypothalamus with an antagonist impairs peripheral glucose metabolism and leads to insulin responsiveness. CCL5-CCR5 activity in hypothalamus thus may be critical for insulin sensitivity regulation in DM. Our findings also provide a new link between chemokine CCL5 and its receptor CCR5 to neuronal function and DM.

## Results

### CCR5 in hypothalamic ARC neurons is associated with neuronal energy metabolism regulation

**Figure 1:**
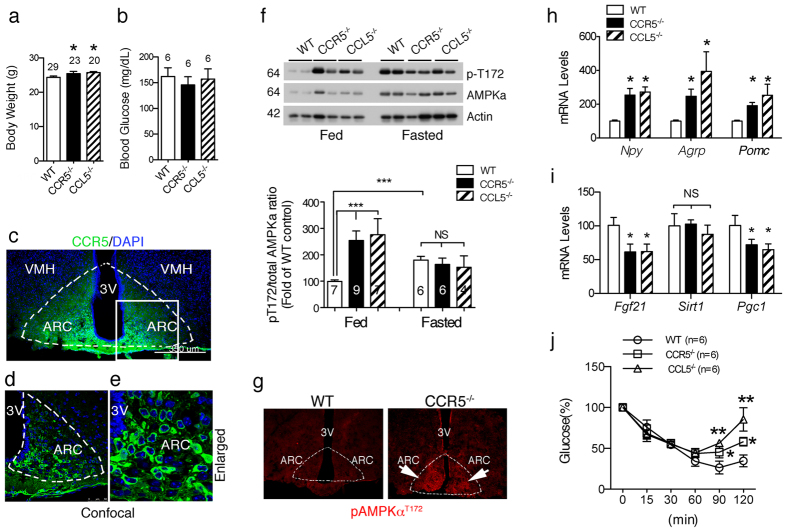
Neuronal energy utilization and insulin regulation was impaired in mouse hypothalamic arcuate nucleus (ARC) without CCR5/CCL5. The body weights (**a**) and fasting blood glucose (**b**) in WT, CCR5^−/−^, and CCL5^−/−^ mice. (**c**) CCR5 antibody labeled the neurons in ARC region (Dash circled triangles). Confocal images of CCR5 in ARC neurons are as in (**d**), and enlarged in the (**e**) (Green: CCR5; Blue: DAPI labeled nucleus. 3V: third ventricle, ARC: arcuate nucleus, VMH: ventromedial hypothalamus). (**f**) The energy sensor–AMPKα activities were increased in WT mouse hypothalamus after 8 hr fasting; mice without CCR5 or CCL5 expressed high levels of pAMPKα under both fed and fasting conditions. Actin was used as the internal control. The quantifications are presented as mean ± SEM. (**g**) Phospho-AMPKα^T172^ immunoreactivity (red) was enriched in the ARC region of the hypothalamus in CCR5^−/−^ mice. The mRNA levels of appetite and body energy regulatory genes including NPY, AgRP, POMC (**h**), FGF-21, SIRT-1 and PGC-1 (**i**) in mouse hypothalamus were analyzed by q-PCR assay (n = 6 in each groups). The insulin tolerance test is shown (**j**) Data are presented as mean ± SEM values (*p < 0.05; **p < 0.01; ***p < 0.001, compared to WT, by *t-test.* ND: no significant difference).

To understand the basic role of CCL5 and CCR5 in energy metabolism, we studied both CCR5^−/−^ and CCL5^−/−^ mice compared to age matched WT mice at 3~4 months of age. The body weights of CCR5^−/−^ and CCL5^−/−^ mice were within the normal range and only slightly higher than WT mice (Fig. 1a). The fasting glucose levels of three types of mice were no different than previously reported^17^,^18^ (Fig. 1b). We next examined the distribution of CCR5 and CCL5/RANTES in the brain by immunostaining. CCR5 immunoreactivity was specifically enriched around ARC region neurons (Fig. 1c-e) only and ligand-CCL5/RANTES was expressed in both ARC and VMH (ventromedial hypothalamus) region as reported^23^ (Supl. Fig. 2b), which suggests that CCL5 and CCR5 might form an antocrine loop in ARC. AMPKα activity in hypothalamus, manifested as AMPKα-Thr^172^ phosphorylation, was increased after 8 hr of fasting in WT mice; however, AMPKα activity was high in CCR5^−/−^ and CCL5^−/−^ mice under both feeding and fasting conditions (Fig. 1f). Immunostaining of phosphor-AMPKα^T172^ confirmed the specifically increased activation of AMPKα in the CCR5^−/−^ mouse ARC region (Fig. 1g). Control staining by omission of the primary antibodies are shown in Supl. Fig. 1a, b. These data suggest that CCR5 and CCL5 in hypothalamus may participate in hypothalamic energy or appetite regulation. Using quantitative PCR (q-PCR), we further examined appetite-related gene expression, such as neuropeptide Y (NPY), AgRP, and POMC, whose mRNA levels were all increased in CCR5^−/−^ and CCL5^−/−^ mouse hypothalamus with regular diet (Fig. 1h). These enhancements, in both appetite-suppressing and stimulating pathways, balanced the level of food intake in CCR5^−/−^ and CCL5^−/−^ mice which explained why food intake and caloric intake were not altered in knockout (KO) mice in both our study (not shown) as well as in previous reports^17^,^18^. In addition, Fibroblast growth factor 21 (FGF21), Sirtuin 1 (SIRT-1) and Peroxisome proliferator-activated receptor gamma coactivator 1-alpha (PPAGRC1, PGC-1) play important roles in the control of energy balance and glucose metabolism by acting in the brain to increase energy expenditure and to improve insulin sensitivity^44^,^45^,^46^,^47^,^48^,^49^,^50^. The energy and insulin signaling regulatory related genes FGF-21 and PGC-1 were significantly reduced in CCR5^−/−^ and CCL5^−/−^ mouse hypothalamus as shown by PCR (Fig. 1i). The insulin response was impaired in CCL5^−/−^ and CCR5^−/−^ mice using the insulin tolerance test (ITT) (Fig. 1j). Our findings thus suggest that CCL5-CCR5 signaling in hypothalamic ARC contributes to insulin-related energy homeostasis in hypothalamic neurons.

### CCR5/CCL5 deficiency disrupts insulin signaling through impaired de-phosphorylation of IRS-1 Serine 302 in hypothalamus

**Figure 2:**
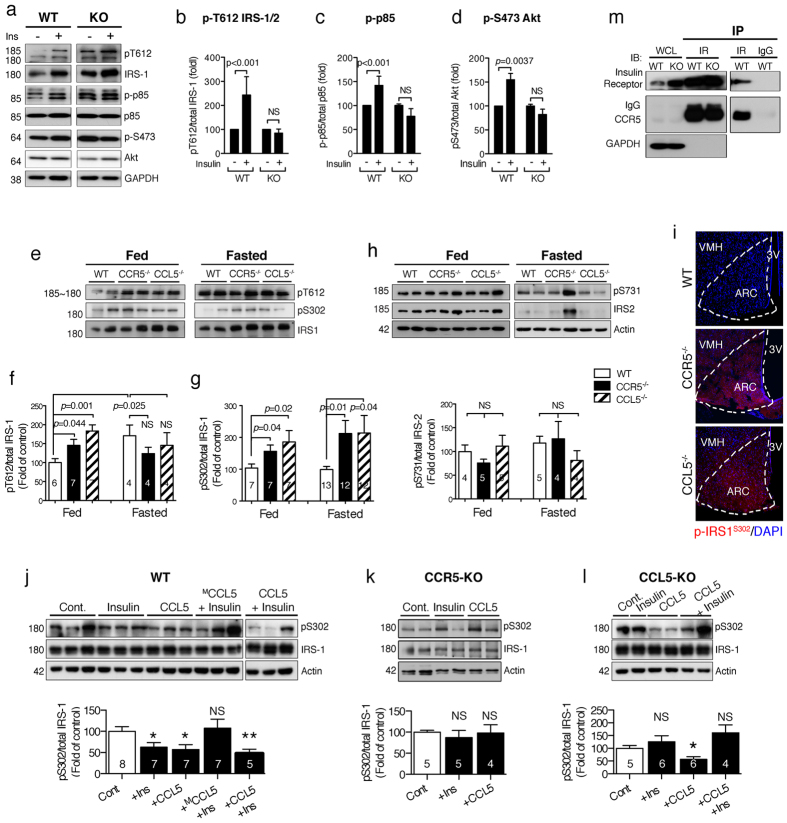
CCR5 association with the insulin receptor participates in hypothalamic insulin signal activation. (**a–d**) The hypothalamus tissues from WT and CCR5^−/−^ mice were stimulated with insulin (10 nM) *ex vivo*. (**a**) The immunoblot of the insulin signaling molecules as IRS-1, PI3K–p85 and Akt in hypothalamus were activated by insulin in WT but not in CCR5^−/−^ (KO) mice. The quantification results are in (**b**) for phosphor-IRS-1^T612^, (**c**) for phosphor-P85, and (**d**) for phosphor-Akt^S473^. The phosphorylation status of insulin substrate–IRS-1 and IRS-2 under feeding and fasting were analyzed. (**e–g**) The activated form of IRS-1 phospho-Tyr612 and inhibitory form of phospho-Ser302 were increased in both fasted and non-fasted CCR5^−/−^ and CCL5^−/−^ mouse hypothalamus. (**h**) IRS-2 activities in CCR5^−/−^ and CCL5^−/−^ were not different from WT under both non-fasting and fasting conditions. (**i**) Immunoreactivity of p-IRS-1^S302^ (red) was detected in the ARC region of CCR5^−/−^ and CCL5^−/−^ mice (Nucleus: blue) (3V: third ventricle, ARC: arcuate nucleus, VMH: ventromedial hypothalamus). (**j–l**) Hypothalamus tissues isolated from 8 hr fasted mice following stimulation with: (1) PBS for 5 min as control (Cont.), (2) Insulin (Ins, 10 nM) 5 min, (3) CCL5 (10 ng/ml) 5 min, (4) combined insulin and CCL5 (Ins + CCL5) for 5 min, and (5) the CCL5 antagonist–methylated-CCL5 (^M^CCL5, 10 ng/ml) 10 min pretreatment before insulin (^M^CCL5 + Ins). The phosph-IRS-1^S302^ levels under different conditions were analyzed by protein blot. The relative levels of p-IRS-1^S302^ under various treatments in WT mice (**j**), CCR5^−/−^ mice (**k**), and CCL5^−/−^ mice (**l**) are summarized. (*p < 0.05; **p < 0.01 compared to control; NS: no significant difference). (m) Hypothalamic tissues from WT and CCR5^−/−^ (KO) were incubated with insulin receptor (IR) antibody or rabbit-IgG (as control) as indicated and immunoprecipitated with protein-A beads. Whole cell lysate (WCL) and co-immunoprecipiated proteins (IP) were probed with insulin receptor (IR), CCR5 and IRS-1. GADPH was used as loading control.

To validate insulin function in hypothalamus and avoid peripheral interference, such as from leptin, hypothalamic tissues from WT and CCR5^−/−^ mice were isolated in order to perform *ex vivo* insulin stimulation. Animals were fasted for 8 hr before sacrifice to lower the insulin level. The insulin downstream signaling molecules including IRS-1/2 (Fig. 2a, b, and Supl. Fig. 3a), PI3K-p85 (Fig. 2a, c, and Supl. Fig. 3b), and Akt (Fig. 2a, d, and Supl. Fig. 3a) activities were increased in WT hypothalamic tissues, but not in CCR5^−/−^ hypothalamus, after insulin (10 nM) treatment. However, the insulin receptor can be activated in both WT and CCR5^−/−^ hypothalamic tissues upon insulin exposure (Supl. Fig. 3c) which suggests CCR5 might participate to the downstream regulation of the insulin signal.

Insulin binding to the insulin receptors activates the downstream–IRS-1 and IRS-2 moieties that act as the regulatory molecules for insulin signaling. We thus examined the activation of IRS-1 and IRS-2 in WT and knockout mice under fasting or feeding conditions. The activation of IRS-2 was similar among these three types of mice (Fig. 2h). We found that both the active form of IRS-1 (phosphorylation of IRS-1/2 at Tyr612 site, p-IRS-1/2^T612^) (Fig. 2e, f) and the inhibitory form of IRS-1 (phospho-IRS-1^Ser302^, p-IRS-1^S302^) (Fig. 2e, g) were increased in CCR5^−/−^ and CCL5^−/−^ mouse hypothalamus under feeding and fasting conditions. The mRNA levels of IRS-1 and IRS-2 in hypothalamus were not changed among WT, CCR5^−/−^ and CCL5^−/−^ mice (Supl. Fig. 4a, b). The increase of p-IRS-1^S302^ in CCR5^−/−^ and CCL5^−/−^ mouse ARC neurons under feeding was confirmed by immunostaining (Fig. 2i).

The phosphorylation of Ser^302^ in IRS-1 which causes a physical dissociation of IRS-1 from the insulin receptor (IR) has a strong inhibitory function in insulin signaling and interrupts insulin signal transmission^33^. We thus studied the de-phosphorylation of IRS-1^S302^ *ex vivo* under (1) saline for 5 min as control; (2) insulin (10 nM), 5 min; (3) CCL5 (10 ng/ml), 5 min; (4) insulin and CCL5 together for 5 min, or (5) 10 min pretreatment with an antagonist Met-CCL5 (^M^CCL5, 10 ng/ml) followed by insulin stimulation for another 5 min. The dosage of CCL5 was similar to that in previous studies^1^,^2^,^51^. The level of phosphor-IRS-1^S302^ was reduced in isolated WT hypothalamic tissue after 5 min insulin stimulation (Fig. 2j). Activation of CCR5 by CCL5 treatment also reduced the level of phosphor-IRS-1^S302^ in WT and CCL5^−/−^ animals (Fig. 2j, l). Co-treatment with insulin and CCL5 showed a synergistic effect on the p-IRS-1^S302^ reduction in WT hypothalamus (Fig. 2j). The CCL5 antagonist-^M^CCL5 fully abolished the reduction of p-IRS-1^S302^ by insulin in WT (Fig. 2j) as well as in CCR5 (Fig. 2k) and CCL5 (Fig. 2l) deficient hypothalamus. Interestingly, the synergistic effect of CCL5 and insulin on the reduction of p-IRS-1^S302^ was impaired in CCL5^−/−^ hypothalamus (Fig. 2l). This might be due to the long-term resistance to insulin in CCL5^−/−^ tissue. The other serine phosphorylation sites such as Ser^612^, Ser^633/639^, and Ser^1101^ were not affected in CCR5^−/−^ hypothalamus (Data not shown). CCR5^−/−^ mice have a functional mutation knockout, in which the C-terminal of CCR5 is mutated with a loss of function^13^. Co-immunoprecipiation of the insulin receptor (IR) with hypothalamus tissue lysates shows CCR5 protein in WT and CCR5^−/−^ lysates (Fig. 2m). Taken together, we find CCR5/CCL5 signaling is involved in insulin signaling regulation through regulating IRS-1^Ser302^ phosphorylation in hypothalamus and the C-terminal domain of CCR5 is essential for this interaction.

### CCR5 deficiency desensitizes insulin signaling by increasing p70 S6 kinase-mediated serine 302 phosphorylation of IRS-1

**Figure 3:**
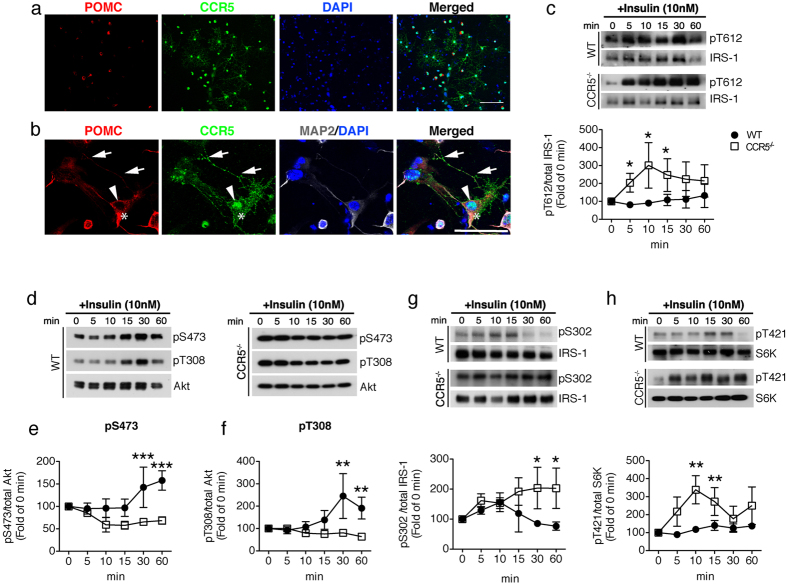
CCR5 expression in hypothalamic neurons participates in insulin signal activation *in vitro*. (**a, b**) Enriched hypothalamic neuron cultures were labeled with CCR5 antibody (green), POMC (red) for hypothalamic specific neurons, and MAP2 (gray) as a neuronal marker. DAPI labeled nucleus in blue. Lower panels are magnified images of primary neurons. Arrows point to neurite and soma expressions of CCR5 and arrowheads point to the nuclear localization of CCR5. Asterisks point to POMC enrichment. Hypothalamic neurons cultured from WT and CCR5^−/−^ mice were stimulated with insulin (10 nM) and cell lysates collected after 0, 5, 10, 15, 30, and 60 min. The insulin signaling molecule p-IRS-1^T612^ (**c**) was markedly activated in CCR5 deficient hypothalamic neurons; the activation of downstream Akt shown as as S473 (**d, e**), and T308 phosphorylation (**d, f**) were impaired in CCR5 deficient neurons. The inhibitory phosphorylation of insulin signals shown as IRS-1Ser302 (**g**) and phospho-T421 of S6 kinase (**h**) were increased in CCR5 deficient neurons after insulin stimulation. Each group shows 4 independent repeated experiments. (*p < 0.05; **p < 0.01; ***p < 0.001 between WT and CCR5^−/−^ groups by Two-way ANOVA test.) Scale bar in a = 100 μm; in b = 50 μm.

Long-term exposure to high blood levels of insulin can cause insulin receptor desensitization and negative regulation of IRS function; whether CCR5 participates in insulin desensitization or IRS-1^Ser302^ activation is important to elucidate. We further investigated the role of CCR5 in insulin signaling using primary cultured hypothalamus neurons from WT and CCR5^−/−^ mice. Immunostaining of CCR5 revealed a vesicular and granular pattern of CCR5 in neuronal soma and which extended into both axons and dendrites (Fig. 3a, b). Co-labeling with POMC (red) and MAP2 confirmed the expression of CCR5 in the enriched primary hypothalamic neuron cultures (Fig. 3b). CCL5/RANTES is expressed in POMC neurons in the culture (Supl. Fig. 2c) which confirms the findings in tissue and supports that CCL5-CCR5 may form an antocrine loop in ARC. We then tested CCR5-mediated insulin signaling in these primary neurons. The activation of IRS-1 (p-IRS-1/2^T612^) was markedly increased after 5 min insulin (10 nM) treatment in cultured CCR5^−/−^ hypothalamic neurons; in contrast, the activation of IRS-1 was only slightly enhanced after 15 min in cultured WT hypothalamic neurons (Fig. 3c). Akt activity was increased after 30 min in insulin treated WT hypothalamic neurons (Fig. 3d, e, f) and the inhibitory signal via p-IRS-1^S302^ was reduced in parallel (Fig. 3g). In contrast, Akt was unable to be activated (Fig. 3d–f) and the inhibitory signal via p-IRS-1^S302^ was increased in CCR5 deficient hypothalamic neurons after 30 min of insulin administration (Fig. 3g), as was also shown with our *ex vivo* studies in Fig. 2.

**Figure 4:**
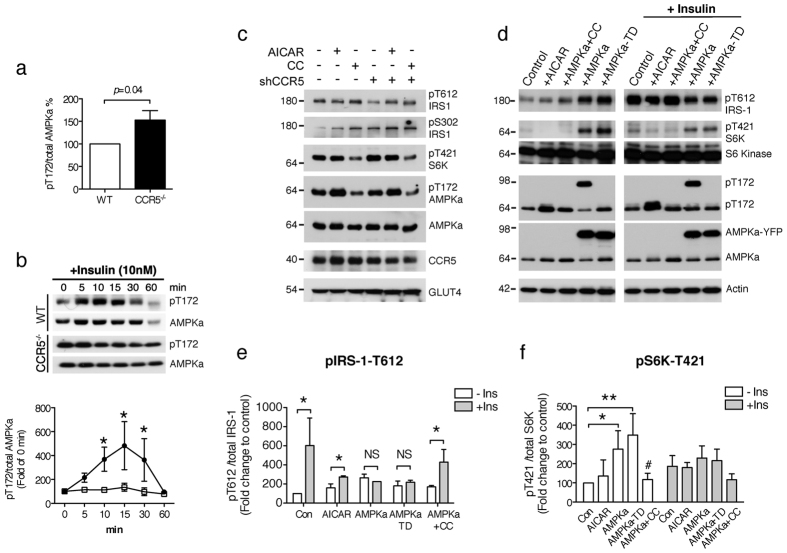
Sustained activation of AMPKα and reduction of CCR5 evokes S6 kinase activity and insulin responsiveness. (**a**) CCR5^−/−^ hypothalamic neurons have higher AMPKα activity–phosphor-AMPKα^T172^ in culture (N = 4). (**b**) Insulin stimulation evoked a transient increase of AMPKα activation in WT hypothalamic neurons (N = 3 in each groups). (**c**) Reduction of CCR5 by shRNA in N2A cells and control N2A cells were treated with AICAR or Compound C (CC) for 15 min. The activities of AMPKα, S6K, and IRS-1, CCR5 and GLUT4 expression levels were analyzed by protein blot. The phosphorylation of IRS-1^T612^ was reduced in CCR5 knockdown cells whereas p-IRS-1^S302^ and p-S6K^T421^ were increased. S6K activity was not affected by AICAR treatment but was reduced by Compound C. (d) N2A cells treated with either AICAR or by overexpression AMPKα and AMPKα-TD (AMPKα-T172D) had higher levels of phospho-AMPKαT172. (**d, e**) The activation of IRS-1/2 by insulin was diminished in cells overexpressing AMPKα and AMPKα-TD, and improved by CC. (**d, f**) S6 kinase activity was enhanced by constitutively expressed AMPKα and AMPKα-TD. (N = 3; *p < 0.05; **p < 0.01 compare to control; ^#^p < 0.05, compared to AMPK expressing cells. NS: not significant difference).

P70-S6 kinase plays an important role as a negative regulator of insulin signaling through serine phosphorylation of IRS-1, and is known to be stimulated by the active form of AMPKα. We found that P70 S6 kinase was markedly activated (p-S6K^T421^) after 5 to 10 min of insulin treatment in neurons lacking CCR5 (Fig. 3h). Elevated AMPKα activity was seen not only in CCR5 knockout hypothalamic tissue but also in cultured CCR5 knockout hypothalamic neurons (Fig. 4a). A transient increase in p-AMPKα^T172^ was evoked by insulin in WT hypothalamic neurons (Fig. 4b) and a constant high level of p-AMPKα^T172^ was seen in CCR5 deficient neurons (Fig. 4a, b). To further evaluate the correlations among CCR5, AMPKα and S6K in insulin activity regulation, we next examined Neuro2A cells transfected with CCR5-shRNA to reduce CCR5 expression. The phosphorylation of S6K and IRS-1^Ser302^ was increased when CCR5 was knocked down (Fig. 4c) and the active form of IRS-1^T612^ was reduced. Short-term treatment with AICAR (15 min), a specific activator of AMPKα, increased AMPKα phosphorylation but not that of S6K; the AMPK inhibitor-compound C (CC) treatment reduced both AMPKα and S6K activation (Fig. 4c). Thus, transient activation p-AMPKα is not sufficient to impair S6K activation, but a reduction of AMPKα can reduce S6K activation. We further tested the insulin response with AMPK activity. The activation of p-IRS-1/2^T612^ was reduced by AICAR treatment but was still higher than in untreated cells (Fig. 4d-f). AMPKα and AMPKα active form–AMPKα-TD (p-AMPKα^T172^) plasmids were next transfected into cells to increase intracellular AMPKα constitutively (Fig. 4d), which increased S6K activity as well (Fig. 4d, f). Insulin activation of p-IRS-1/2^T612^ was impaired in the AMPKα and AMPKα-TD expressing cells (Fig. 4d, e). Additionally, compound C treatment in AMPKα expressing cells reversed the impaired activation of p-IRS-1/2^T612^ (Fig. 4d, e). Thus, sustained activation of AMP Kinase is a key factor that impairs insulin signaling activation and IRS-1^Ser302^ de-phosphorylation.

This aberrant increase of p-S6K^T421^ was also evidenced in fed CCR5^−/−^ mouse ARC neurons specifically (Supl. Fig. 5b) but not in WT mice (Supl. Fig. 5a) using immunostaining analysis. The activation of Akt–(p-Akt^S473^) was enhanced in WT mice in but not in CCR5^−/−^ mice ARC (Supl. Fig. 5d, c). These *in vivo* findings are consistent with the *in vitro* studies.

### Activating CCR5 promotes GLUT4 membrane translocation

**Figure 5:**
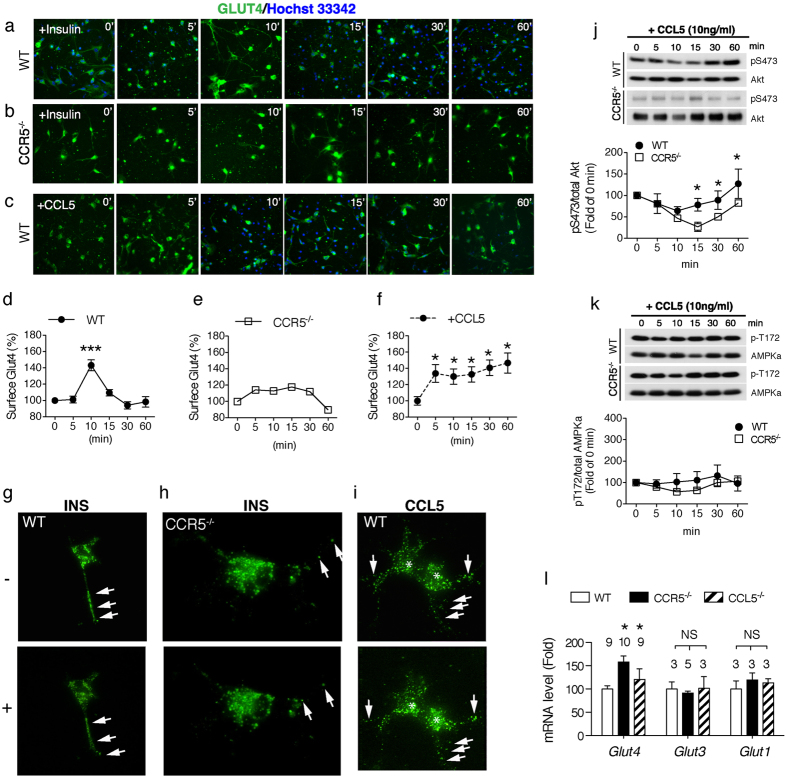
Elevated CCR5 increased Akt Ser473 phosphorylation and GLUT4 plasma membrane expression in hypothalamic neurons. (**a–c**) The membrane glucose transporter-4 (GLUT4) was detected by a specific antisera and a green fluorescence probe. The plasma membrane expression of GLUT4 in WT and CCR5^−/−^ neurons was measured by the green fluorescence intensity on membranes after insulin or CCL5 stimulation. (**d–f**) The changes in membrane GLUT4 expression by insulin and CCL5 were converted into relative fluorescence intensity (N = 4, 34~110 cells were quantified at each time point). Hoechst 33342 labeled the un-penetrated cell nucleus. GLUT4 membrane translocation was quickly stimulated by insulin after 5–15 min in WT hypothalamic neurons (**a, d**); the changes in GLUT4 on membranes were more gradual in CCR5^−/−^ neurons (**b, e**). CCL5 increased GLUT4 membrane expression (**c, f**). Representative images of GFP-GLUT4 neuritic movement upon insulin in WT (**g**) and CCR5^−/−^ hypothalamic neuron (**h**). (**i**) The GFP-GLUT4 neuritic movement upon CCL5 in WT hypothalamic neurons. Arrows point to the GFP-GLUT4 puntas on neurites. Asterisks label the surface enrichment of GFP-GLUT4. Akt Ser473 phosphorylation by a CCR5 dependent mechanism (n = 3) (**j**). (**k**) AMPKα activity in hypothalamus neurons was independent of CCL5 and CCR5 (n = 4). (**l**) The expression of the insulin-related glucose transporter gene- GLUT4 was increased in CCR5^−/−^ and CCL5^−/−^ mouse hypothalamus but the insulin independent neuronal glucose transporter GLUT3 and astroglial glucose transporter GLUT1 in mouse hypothalamus were not changed, using q-PCR analysis. (***p < 0.0001, *p < 0.05 by one-way ANOVA in **d**, **f**; *p < 0.05, by Two-way ANOVA in **g**; *p < 0.05, by *t-test*, NS: no significant difference in **l**).

What causes the marked activation of AMPKα in CCR5 deficient hypothalamus neurons remains unclear and the role of AMPKα activation by insulin is also unknown. AMPK can be activated by many factors and by impaired cellular energy status such as a high AMP/ATP ratio. Neurons in the brain are not the major target for glucose uptake in response to insulin; however, hypothalamic ARC neurons express large amounts of GLUT4^52^ and CCL5 is found to increase glucose uptake in T-cells and to reduce food intake. We thus further analyzed the influence of CCR5 on GLUT4 plasma membrane translocation in primary hypothalamic neurons. The expression of membrane GLUT4 was detected using specific antisera with a fluorescence probe. GLUT4 membrane expression was transiently activated over 5 to 15 min with a peak at 10 min after insulin stimulation in WT neurons (Fig. 5a, d). This phenomenon was seen earlier than the activation of AMPKα and Akt described above; a slight increase in membrane GLUT4 was evident in CCR5 deficient neurons (Fig. 5b, e). The total amount of GLUT4 in each sample was not different with immunolabeling (Data not shown) and protein blot (Supl. Fig. 6a-b). CCL5 activation of CCR5 increased GLUT4 membrane translocation after 5 min (Fig. 5c, f) which was sustained for 60 min. We also introduced GFP conjugated GLUT4 plasmids into hypothalamic neurons. Live recoding of GLUT4 movement upon insulin and CCL5 was carried out. Insulin stimulated GLUT4-GFP neurite movement in WT hypothalamic neurons (Supl. Movie-1), whereas little GLUT4-GFP movement was seen upon insulin stimulation in CCR5^−/−^ hypothalamic neurons (Supl. Movie-2). The representative images of GFP-GLUT4 movement upon insulin in WT and CCR5^−/−^ hypothalamic neuron are seen in Fig. 5 g, h. CCL5 administration induced abundant GLUT4-GFP punta neuritic transport in WT (Fig. 5i and Supl.Movie-3). This is consistent with the GLUT4 membrane staining results noted above. CCL5 increased Serine473 phosphorylation of Akt but not tyrosine308 (not shown) in conjunction with CCR5 expression (Fig. 5j). However, CCL5 did not increase AMP-Kinase activity (Fig. 5k). These changes are different from those of insulin signaling and suggest that CCL5/CCR5 signaling of GLUT4 regulation in hypothalamus has a specific function. The mRNA levels of GLUT4 but not the other insulin-independent glucose transporters-GLUT1 and GLUT3 (Fig. 5l) were compensatorily increased in CCR5 and CCL5 deficient mouse hypothalamus.

Parallel studies with cerebral cortex and primary cortical neurons were also carried out. We found a different regulation of IRSs and insulin signaling in mouse cerebral cortex (Supl. Figs 7–8). The mRNA of IRS-1 was reduced in cortex of both types of KO mice, whereas IRS-2 was increased in CCL5^−/−^ cortex (Supl. Fig.7a, b). The transcription levels of GLUT3 and GLUT1 in CCR5^−/−^ mouse cerebral cortex were not changed, whereas the level of GLUT4 was increased in CCL5^−/−^ but not in CCR5^−/−^ cerebral cortex (Supl. Fig.7e). These data suggest that CCL5 may be an important contributor to cortical neuron energy supply via different receptors than CCR5; in contrast CCR5 predominately contributes to hypothalamic neuron energy regulation. The activation of IRS-1 and Akt were not changed by either insulin or CCL5 administration in primary cortical neurons, which contain a lower level of GLUT-4 in cortex (Supl. Fig. 8a–h). These data further support the specific role of CCR5 in hypothalamus. Taken together, we have thus identified a specific role for CCR5 in insulin signaling regulation and glucose uptake in hypothalamus.

### CCR5 modulation of insulin signals in hypothalamus contributes to systemic glucose metabolism and insulin resistance

**Figure 6:**
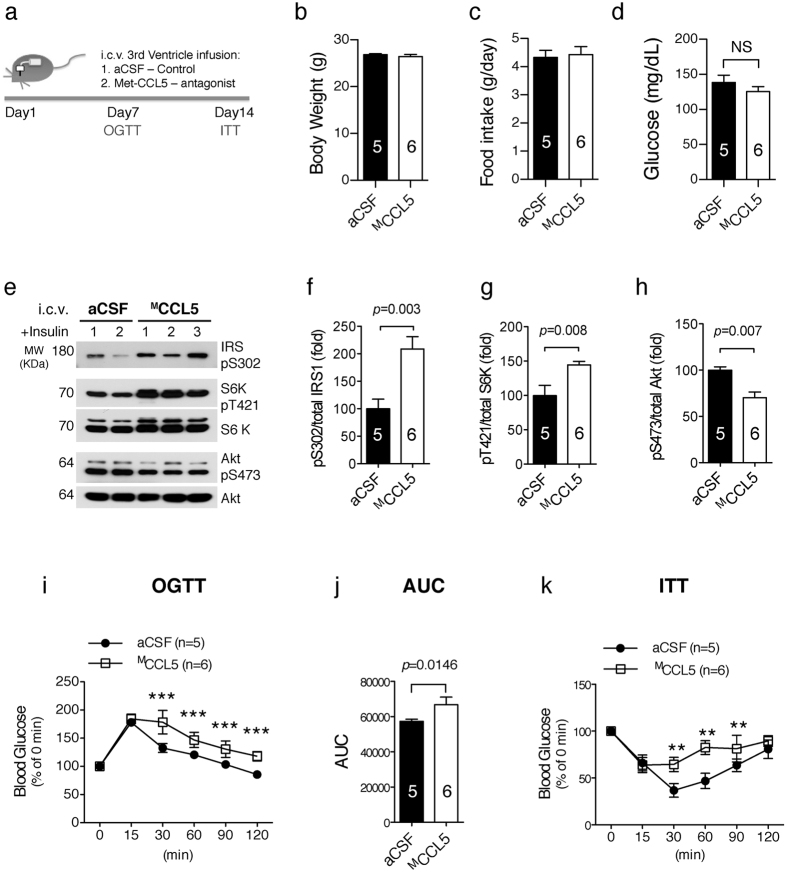
Blocking hypothalamic CCR5 with the antagonist-^M^CCL5 induced glucose metabolism impairment and insulin resistance peripherally. (**a**) Wildtype mice received 3^rd^ ventricle ICV infusion with aCSF as control or a CCL5 antagonist–Met-CCL5 (^M^CCL5) to block CCR5 over two weeks. The body weight (**b**), daily food intake (**c**) and fasting blood glucose (**d**) in two groups of mice were similar. (**e–h**) The activation of insulin signaling manifested as phospho-Akt^S473^ was reduced in ^M^CCL5-treated mouse hypothalamus; the inhibitory signals as phosphor-IRS-1^S302^ and phosphor-S6K^T421^ were increased in mice receiving the antagonist-^M^CCL5. OGTT (oral glucose tolerance test) (**i**), the AUC (area under curve) of OGTT (**j**), and ITT (insulin tolerance test) (**k**) were impaired in mice treated with antagonist ^M^CCL5. (n = 5~6 in each groups, ***p = 0.001, **p = 0.0015, by Two-way ANOVA in **i, k**).

We subsequently investigated the contribution of CCR5-mediated hypothalamic signaling to systemic insulin sensitivity and glucose metabolism. We found that antagonist ^M^CCL5 pretreatment blocks hypothalamic insulin signal activation *ex vivo* (Fig. 2j). WT mice were intracerebroventricularly (ICV) infused with aCSF (artificial cerebrospinal fluid) or ^M^CCL5 (10 ng/ml) into the 3^rd^ ventricle for 2 weeks using an Alzet pump system. An oral glucose tolerance test (OGTT) and an insulin tolerance test (ITT) were carried out after 7~14 days infusion (Fig. 6a). The body weights, daily food consumption, and fasting blood glucose of two groups of mice were not different as with whole body knockout animals (Fig. 6b–d). The insulin signal activation in hypothalamus measured as p-Akt^S473^ was reduced in ^M^CCL5-infused mice (Fig. 6e,h), whereas the inhibitory signaling molecules, p-IRS-1^S302^ and p-S6K^T421^, were increased (Fig. 6e,f,g). The glucose metabolism rate was reduced in the ^M^CCL5 infused mice compared to the aCSF group, manifested as increases in the OGTT time (Fig. 6i) and the area under the OGTT curve (AUC) (Fig. 6j). Blood glucose was slightly reduced but quickly returned in ^M^CCL5-infused mice after 15~90 min with intraperitoneal insulin injection (Fig. 6k); in contrast, blood glucose was markedly reduced by insulin but returned after 120 min in aCSF group mice. These results suggest that insulin resistance exists in mice after ^M^CCL5 brain infusion. Together, the data suggest that blocking CCR5 receptors in hypothalamus interferes with hypothalamic insulin signaling and causes peripheral glucose intolerance and insulin resistance.

## Discussion

**Figure 7:**
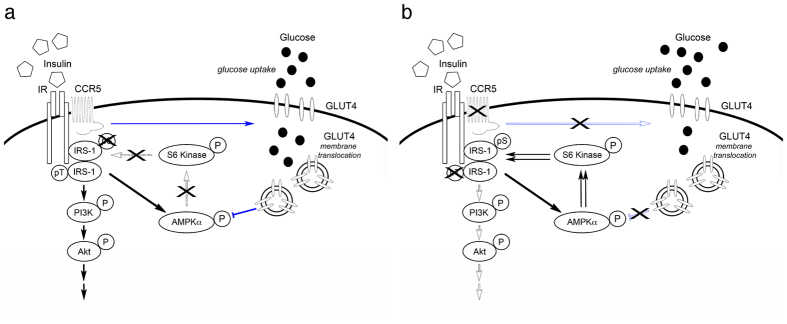
Summary diagram of CCR5’s role in hypothalamic insulin signal regulation. CCR5 activation plays a “checkpoint” role during insulin activation in hypothalamus. (**a**) Activating CCR5 promotes GLUT4 membrane translocation and glucose uptake in hypothalamic neurons which reduces AMPKα activation and promotes insulin signaling downstream by PI3K and Akt activation. (**b**) GLUT4 membrane translocation upon insulin exposure is reduced without CCR5; insufficient energy and increased AMPKα activity enhances S6 Kinase phosphorylation of IRS-1 at the serine-302 site and blocks insulin downstream signaling.

There are three major findings in the current study. First, CCR5 is highly expressed in hypothalamic ARC neurons and associates with the insulin receptor for insulin signal regulation in hypothalamic neurons. Second, we found that CCL5 activation of CCR5 induces GLUT4 membrane translocation for glucose uptake, which blocked negative regulation of the insulin signal by AMPKα and S6 Kinase serine phosphorylation of IRS-1 (Fig. 7a); the loss of CCR5 caused aberrant activation of AMPKα and a constant phosphorylation of IRS-1 ser302 which, in turn, led to insulin resistance (Fig. 7b). Third, CCR5 and CCL5 regulatory effects on insulin signaling in hypothalamus contribute to peripheral insulin resistance and glucose intolerance. Thus, CCR5-CCL5 activation promotes insulin signaling in hypothalamic neurons and contributes to systemic insulin responsiveness.

The impact of CCR5 in the development of obesity and associated metabolic abnormalities is under debate. Kitade’s and Kennedy’s studies have different findings about the role of CCR5 in high fat diet-induced DM and obesity^17^,^18^. The interaction between central hypothalamic and peripheral immune and energy storage systems (adipocyte and hepatocyte) are complex. Here, we used the same CCR5^−/−^ mice as Kennedy’s group did, and additionally used CCL5^−/−^ mice to address the functions of CCL5-CCR5. Our studies utilized *ex vivo* stimulation and *in vitro* primary neuron cultures to reduce complex effects. We demonstrated that the activation of insulin receptors and IRS-1/2^T612^ was functional, and that the impaired de-phosphorylation of IRS-1^S302^ was the major cause of insulin signal impairment. Other phosphorylation sites such as serine 612, serine 1101 and serine 636/639 were also investigated and were not affected in cultured hypothalamic neurons and tissues (Data not shown). Another insulin receptor downstream signal, the Erk pathway in hypothalamus neurons, has opposite responses to insulin than that shown in hepatocytes^53^ (Supl. Fig. 9 and supl. discussion). This aspect of signaling needs further investigation. However, such differences also indicate that CCR5 and insulin signaling are different amongst various types of cells.

CCR5 is highly enriched in the ARC region in both POMC neurons and non-POMC hypothalamic neurons in culture (data not shown). We found that CCR5 associates with the insulin receptor in hypothalamus and mediates GLUT4 membrane expression. Hypothalamic neurons are glucose responsive with higher protein GLUT4 levels. We postulate that CCR5 may participate in neuronal glucose responsiveness through AMPK activity regulation in hypothalamus. AMPK is essential for cellular energy homeostasis and integrates neurohormonal signals to assess energy balance in the central nervous system. In this study, insulin administration induced a transient activation of AMPK after 5 min and this reached a peak in about 15 min. PI3K and Akt were activated after 15 min. The time course of insulin stimulation of GLUT4 membrane translocation is about 5~15 min earlier than PI3K-Akt and AMPK activation. CCR5 mediation of GLUT4 membrane translocation increases glucose uptake and reduces AMPK activity. Neurons without CCR5 have a sustained higher activation of AMPK that activates S6 kinase (Fig. 4a, c, 3 h). Activating S6K enhanced serine phosphorylation of IRS-1, as serine-302 herein. Phosphor-IRS-1^Ser302^ causes IRS-1 to physically dissociate from the insulin receptor which is a major cause of insulin resistance^33^. Thus, we postulate that CCR5 induction of GLUT4 membrane translocation for glucose uptake in hypothalamic neurons is an energy status regulation step for neuronal glucose and prevents AMPKα and S6K negative regulation at IRS-1. The insulin signal would be activated thus only with sufficient glucose.

The other energy sources in the brain such as lactate and glycogen were not different among WT, CCR5^−/−^, and CCL5^−/−^ animals (not shown). The mRNA levels of lactate shuttles–the neuronal monocarboxylate transporter 2 (MCT-2) were similar in WT, CCR5^−/−^, and CCL5^−/−^ hypothalamus (Supl. Fig. 4c), but were increased in cerebral cortex (Supl. Fig. 7c). The astrocytic monocarboxylate transporter 4 (MCT-4) was increased in CCL5^−/−^ hypothalamus (Supl. Fig. 4d) but was reduced in CCR5^−/−^ cortex (Supl. Fig. 7d).

CCL5 activation of CCR5 directly increased GLUT4 membrane expression after 5 to 60 min without AMP-Kinase activation. This mechanism suggests that CCL5 has a marked effect on glucose uptake in hypothalamus, which explains the reduction in food consumption in rats after ICV administration of CCL5 into brain^22^ and the increased MCT-4 in mice lacking CCL5 (Supl. Fig. 4d). These findings also suggest that GLUT4 and insulin signal regulation is a delicate process and that various cells types might have different specific mechanisms. The role of CCL5/CCR5 in insulin signal regulation is specific through GLUT4. The other CCL5 receptors, CCR1, 3, and 5, levels in hypothalamus and cerebral cortex were all slightly increased in mice lacking CCL5; CCR3 was the only receptor that increased in CCR5 deficient hypothalamus (not shown). We propose that increases in other receptors might be a compensatory mechanism; however, the CCL5-CCR5 mediation of insulin signal regulation in hypothalamus is specific.

How does central CCR5 deficiency alter peripheral insulin responsiveness? AMPK and S6K might contribute to this through a brain–liver axis. In our ICV study, blood glucose was reduced after 60 min in mice with CCR5 antagonists compared to glucose reduction after 30 min in the control aCSF group mice. The ITT test also showed that the reduction in blood glucose was blocked 30 min after insulin injection in mice receiving CCR5 antagonists. Glucose metabolism upon insulin exposure was impaired in mice given a CCR5 antagonist after 30 min. These data suggest that the primary insulin effect within 30 min is functional but a secondary effect of insulin after 30 min involves CCR5 activity in hypothalamus. This blocking effect is sustained over one month; the inhibition of insulin sensitivity gradually recovers due to the half-life of the CCR5 antagonist, indicating that antagonist blocking of the insulin signal is transient and recovery of CCR5 function is able to restore insulin sensitivity and responsiveness. Activating S6K in hypothalamus mediates hepatic insulin resistance^54^; however, the role of CCR5 in peripheral insulin resistance via S6K requires further study.

The autonomic nervous system may also mediate CCL5-CCR5 effects on insulin function. CCL5 is known to increase neuron activity and to modulate glutamate release in neurons^1^,^55^; the autonomic innervation of liver responds to glucose uptake^56^. Recently, sympathetic neuro-adipose connections have been identified^57^. Lack of CCR5-CCL5 might alter glucose metabolism in hepatocytes and reduce lipolysis through the sympathetic neuro-adipose system since the body weight with a regular diet in knockout mice is slightly increased. Additionally, CCR5 and CCL5 actions in peripheral adipose tissue and macrophage infiltration, which also underlie T2DM development, are important. CCR5 deficiency in immune cells are sufficient to prevent impaired glucose intolerance in mice^17^. The role of CCR5 and CCL5 action on peripheral immune cells, inflammation, and hepatocyte glucose metabolism in DM progression require further investigation. The correlation of CCL5-CCR5 signaling with other energy regulation-related molecules such as leptin, and the melanocortin-MC4 receptor system in energy signaling is also worthy of future study. A very recent study in breast cancer cells demonstrated that CCL5 increases glucose uptake and that CCL5-CCR5 interactions can increase cell anabolic metabolism, specially glycolysis, using metabolomic analysis^58^. Thus, CCL5-CCR5 signaling is critical for cellular metabolism regulation in many types of cells. Interestingly, HIV infected patients have a higher prevalence of dyslipidemia, insulin resistance, diabetes, and cardiovascular disease. Both antiviral drugs and the T2DM drug-Metformin improve these metabolic syndromes^59^. HIV (immunodeficiency virus) binds to CCR5 and CXCR4 receptors to infect immune cells and neurons. One may speculate that impaired CCR5 function might be the cause of HIV induced metabolic syndromes.

Defects in insulin mechanisms are also associated with neurodegenerative diseases. Many DM drugs such as metformin, GLP-1 and Exendin-4^60^,^61^,^62^,^63^ are also supportive for neurons and have neuroprotective effects. These are potentially new therapeutic drugs for neurodegenerative diseases and different types of neuronal degenerations^64^,^65^,^66^,^67^. We previously showed that CCL5 has a protective effect on neurons in Huntington’s disease^1^.

In summary, our study showed how the inflammatory chemokine CCL5 and its receptor CCR5 contribute to insulin resistance and glucose metabolism in the hypothalamus. It is suggested that central CCL5-CCR5 signaling might play an important physiological role in regulation of energy metabolism through manipulating hypothalamic energy-control pathways. In clinical studies, CCL5/RANTES and CCR5 in peripheral blood was increased in patients with T2DM and was reduced after insulin therapy^68^. CCL5-CCR5 interactions can increase cell responsively and sensitivity to insulin in our present study. Targeting CCR5 to enhance insulin responsively and sensitivity could thus be a potential treatment for DM in the future.

## Experimental Procedures

### Animals, Physiology and Biochemistry Methods

C57BL/6 mice from the National Laboratory Animal Center were used as wild type (WT). The CCL5^−/−^ (CCL5 knockout) and CCR5^−/−^ (CCR5 knockout)^69^ mice, which are the same as in Kennedy’s study^13^,^18^, were originally from Jackson Laboratory. The mating and genotyping followed Jackson Laboratory protocols. Mice were anesthetized using intraperitoneal injection with Ketamine/Xylazine (Ketamine 50 mg/kg, 10 mg/kg Xylazine) for intracerebral ventricle (i.c.v) infusion surgery. The Alzet pump systems were filled with aCSF or ^M^CCL5 (^Met^RANTES, 335-RM-025, R&D system, 10ng/ml in aCSF) using a brain infusion kit (ALZET^®^ Osmotic Pumps–no. 1002, Cupertino, CA, USA) and placed into 3^rd^ ventricle (bregma: 0.0 mm lateral, 1.3 mm posterior; 5.7 mm ventral). Glucose and insulin tolerance tests were performed at 7, and 14 days after infusion. Following a 6-hr fast, mice were given oral glucose (2.5 g/kg) for the glucose tolerance test (OGTT) or i.p. injection 0.75 U/kg human insulin for the insulin tolerance test (ITT) after a 4-hour fast. Blood glucose levels were obtained immediately before injection (0 min) and at 15, 30, 60, 90, and 120 minutes after injection using a glucose trip (ACCU-CHEK, Roche, Germany). Area under the curve (AUC) values were calculated using Image J. The housing of mice and all the animal studies were performed in the accordance of the protocols approved by the Institutional Animal Care and Use Committees of the Taipei Medical University (Protocol numbers: LAC-2013-0278; LAC-2014-0387). The details of the experiments and studies information are listed in the Supplementary Information.

### Statistics

Data are presented as mean ± SEM values. The variance between samples was analyzed by t-test or two-way ANOVA using Prism (version 5.0) software. *p* values ≤ 0.05 were considered statistically significant.

## Additional Information

**How to cite this article**: Chou, S.-Y. *et al.* CCL5/RANTES contributes to hypothalamic insulin signaling for systemic insulin responsiveness through CCR5. *Sci. Rep.* **6**, 37659; doi: 10.1038/srep37659 (2016).

**Publisher’s note:** Springer Nature remains neutral with regard to jurisdictional claims in published maps and institutional affiliations.

## Supplementary Material

Supplementary Movie 1

Supplementary Movie 2

Supplementary Movie 3

Supplementary Information
