# Supplementary material for: CCL5/RANTES contributes to hypothalamic insulin signaling for systemic insulin responsiveness through CCR5
Source: Sci Rep. 2016 Nov 29;6:37659. doi: 10.1038/srep37659 (PMC5127185; doi:10.1038/srep37659)
Supplement: Supplementary Information [file srep37659-s4.doc]

**Supplementary Files**

**CCL5/RANTES contributes to hypothalamic insulin signaling for systemic insulin responsiveness through CCR5**

Szu-Yi Chou^1,2,3*^, Reni Ajoy^1,2^, Chun Austin Changou^4,5,6^, Ya-Ting Hsieh^1,2^, Yang-Kao Wang^2,7^, Barry Hoffer^1,2,3,6^

(*Corresponding author)

^1^The Ph.D. Program for Neural Regenerative Medicine, College of Medical Science and Technology, Taipei Medical University and National Health Research; ^2^Graduate Institute of Neural Regenerative Medicine, College of Medical Science and Technology, Taipei Medical University, Taipei, Taiwan; ^3^Center for Neurotrauma and Neuroregeneration, College of Medical Science and Technology, Taipei Medical University, Taipei, Taiwan; ^4^The Ph.D. Program for Cancer Biology and Drug Discovery, College of Medical Science and Technology, Taipei Medical University, Taipei, Taiwan; ^4^Integrated Laboratory, Center of Translational Medicine, Taipei Medical University, Taipei, Taiwan; ^5^Core Facility, Taipei Medical University, Taipei, Taiwan; ^6^Department of Neurosurgery, Case Western Reserve University School of Medicine, Cleveland, OH, USA; ^7^Department of Cell Biology­ and Anatomy, National Cheng Kung University Medical College, Tainan, Taiwan

**Inventory of Supplemental Information-**

1. **Supplemental Materials and Methods**
2. **Supplemental Tables**
3. **Supplemental Figures and Legends**
4. **Supplemental Movies**
5. **Supplementary Result and Discussion**
6. **Supplemental References**

**1. Supplemental Materials and Methods**

**Cell Cultures** Hypothalamic and cortical neurons were cultured from C57BL/6 and CCR5^-/-^ embryos at day 16.5 (E16.5)^1^. 4 x 10^5^ cells were placed into 6-well plates; insulin (10 nM, Actrapid, Denmark) and mouse CCL5/RANTES stimulation studies were performed after 10 days in culture. AMPKα and AMPKα-TD plasmids were provided by Dr. Yijuang Chern^2^. GFP conjugated GLUT4 was kindly provided by Professor Samuel Cushman from NIH^3^. CCR5 shRNA was obtained from the Academic Sinica - RNAi Core. Both N2A cells and primary hypothalamic neurons were transfected with indicated plasmids by Lipofectamine 2000 (Invitrogen).

**Hypothalamic Tissue co-Immunoprecipitation and Protein Blot Analyses** Tissues were lysed by PIPA buffer (Millipore) with protease-phosphatase inhibitor (Thermo). The protein blot intensities were quantified by Image J software. In co-immunoprecipitation studies, 100 μg tissues were taken and incubated with protein A Sepharose beads (GE, Healthcare Life Sciences) with the indicated antibody in STET buffer (5% TritonX-100 in STE buffer) at 4°C overnight following standard protocols and then analyzed with Western blotting.

**Antibody Information** The antibodies and titer used in study are as follows: AMPKα (#2603, IB: 1:1000), AMPKα-pT172 (#2535, IB: 1:1000; IHC: 1:200), IRS-1 (#3407, IB: 1:1000), IRS-1 pS302 (#2491, IB: 1:1000, IHC: 1:200), total Akt (#4691, IB: 1:2000), Akt pS473 (#4060, IB: 1:2000; IHC: 1:100), Akt pT308 (#2965, IB: 1:2000), p70 S6 Kinase (#2708, IB: 1:2000), p70 S6Kinase pT421 (#9204, IB: 1:2000; IHC: 1:100), anti- Erk1/2 (#4695, WB:1:5000) anti-phospho-Erk1/2 (#4370, WB:1:5000, IHC:1:100) and insulin receptor (#3025, IP: 1:50, IB: 1:1000) were from Cell Signaling; Actin (1:10000, Millipore) as internal control. Phospho-IRS-1/2 (T612) (sc-17195-R, IB: 1:1000) was from Santa Cruz. CCR5 antibodies were (IHC: 1:200, sc-17833) from Santa Cruz and (IB: 1:1000, NBP1-41434) from NOVUS. Anti-phosphor-insulin receptor Y1361 (ab60946, WB: 1:1000) and POMC (IHC: 1:400, ab32893) was from Abcam, and MAP-2 (1:400, AB5622) was from EMD Millipore. anti-CCL5/RANTES (#478-MR, IHC: 1:100) was from R&D GLUT4 anti-sera (IF: 1:1000) was from Prof. Karin Stenkula (Lund University, Sweden)^4^.

**Brain Slice Preparation and Immunohistochemistry Staining** Brain sections were blocked with 5% normal goat serum in 0.1M PB, followed by primary antibody incubation overnight, and then labeled with biotin-conjugated Rb-IgG with streptavidin-conjugated Alexa-568 (Invitrogen) or with secondary antibodies as Alexa-conjugated Fluro-488, 568. DAPI labeled the nuclei (Sigma-Aldrich). Images were captured using a Leica 6000 microscope.

**Live Image for GLUT4-GFP** For GLUT-4-GFP image acquisition, live cells were imaged with wide-field DeltaVision deconvolution microscope (Applied Precision Inc.) equipped with 60x/1.42 NA oil immersion objective lens. Both microscope and camera were controlled by SoftWorX application software. All images and videos were deconvolved using SoftWorX software (Applied Precision Inc.), and later analyzed with VoloCITY software (PerkinElmer).

**RNA Isolation and Quantitative PCR Analysis** Tissue RNAs were extracted by TRIZOL (Invitrogen) and transcribed into cDNA using the Reverse Transcriptase kit (ABI). Quantitative PCR was performed using ABI StepOnePlus. Primer sequences are listed in Supplementary Table 1. Expression of mRNAs was normalized against β-actin and calculated by the 2^−ΔΔCt^ method.

**2. Supl. Table 1: Primers used in study.**

**
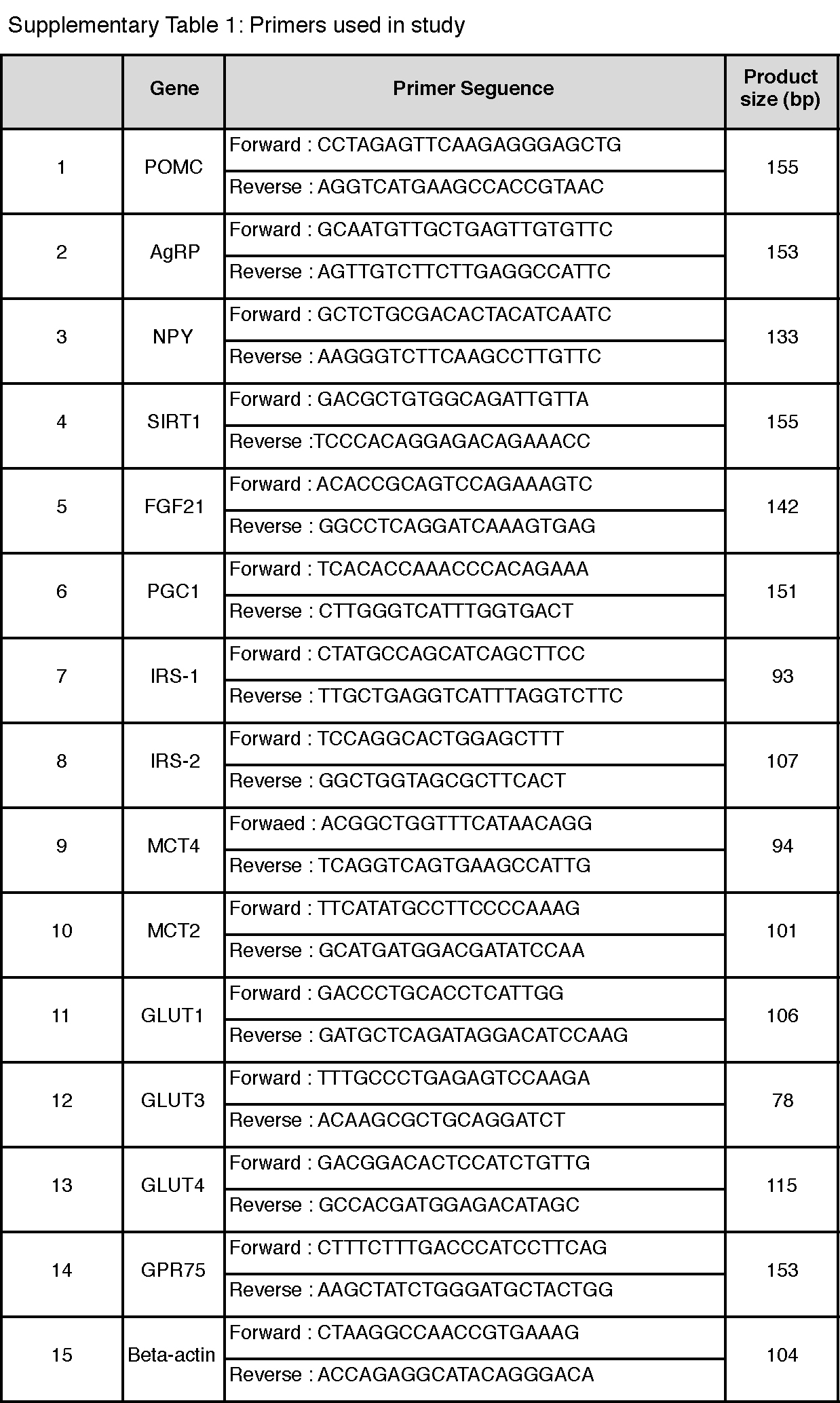
**

**3. Supplemental Figures and Legends**

**
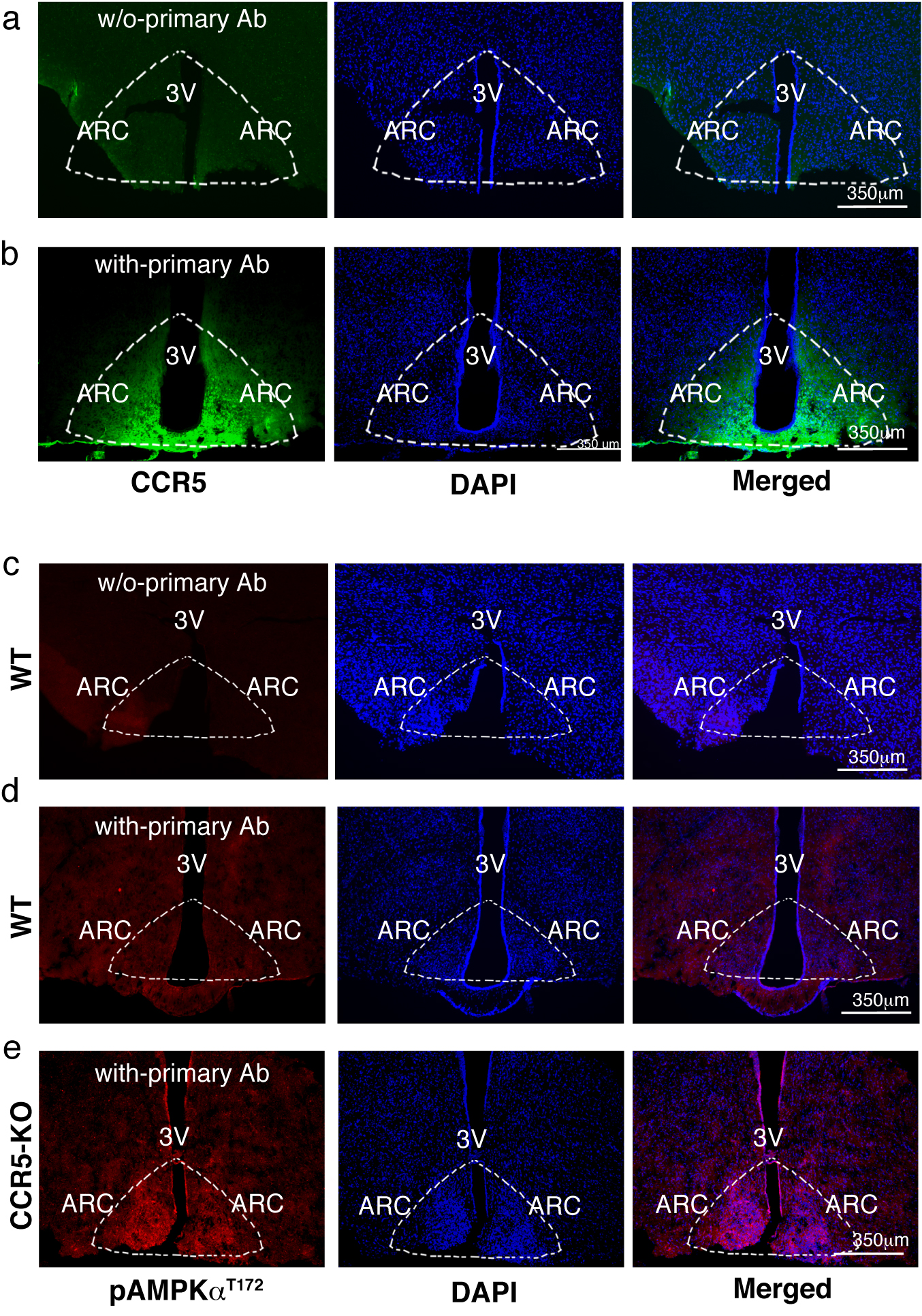
**

**Supl. Fig. 1: The immunostaining of CCR5 and phosphor-AMPKα with and without primary antibodies.** Mouse brain tissues were incubated without (**a**) or with (**b**) CCR5 antibody following the secondary antibody – anti-mouse Alex-488. Labeling was specifically enriched around the ARC region of the brain. For AMPKα activation, brain tissues from wildtype (**d**) and CCR5^-/-^ (**e**) mice were incubated with a specific antibody against phosphor-AMPKαT172 and anti-rabbit IgG conjugated with Biotin following Striptoavidin-Alex-568. Tissues were incubated with secondary antibodies and omission of the primary antibodies was taken for phosphor-AMPKα control staining in (**c**). DAPI labeled the nucleus. The scale bar = 350 μm.


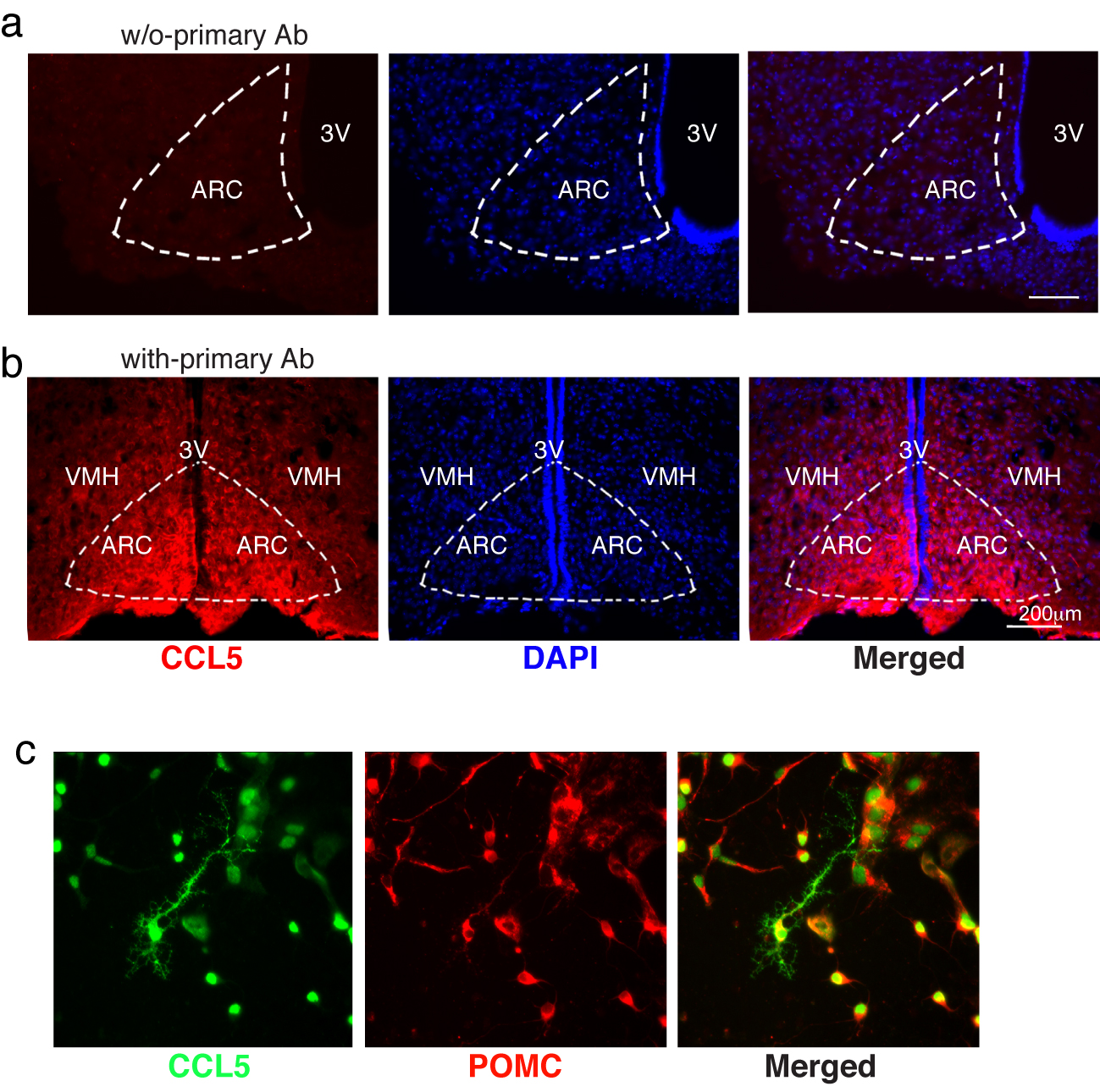


**Supl. Fig. 2: The immunostaining of CCL5/RANTES in mouse brain and primary cultured hypothalamic neurons.** Mouse brain tissues were incubated without (a) or with (b) CCL5/RANTES antibody (red). Both ARC and VMH regions in hypothalamus have strong CCL5/RANTES labeling. The scale bar = 200 μm. CCL5 antibody (green) labeled POMC (red) positive hypothalamic neurons in cultures. Nucleus was labeled by DAPI in blue.


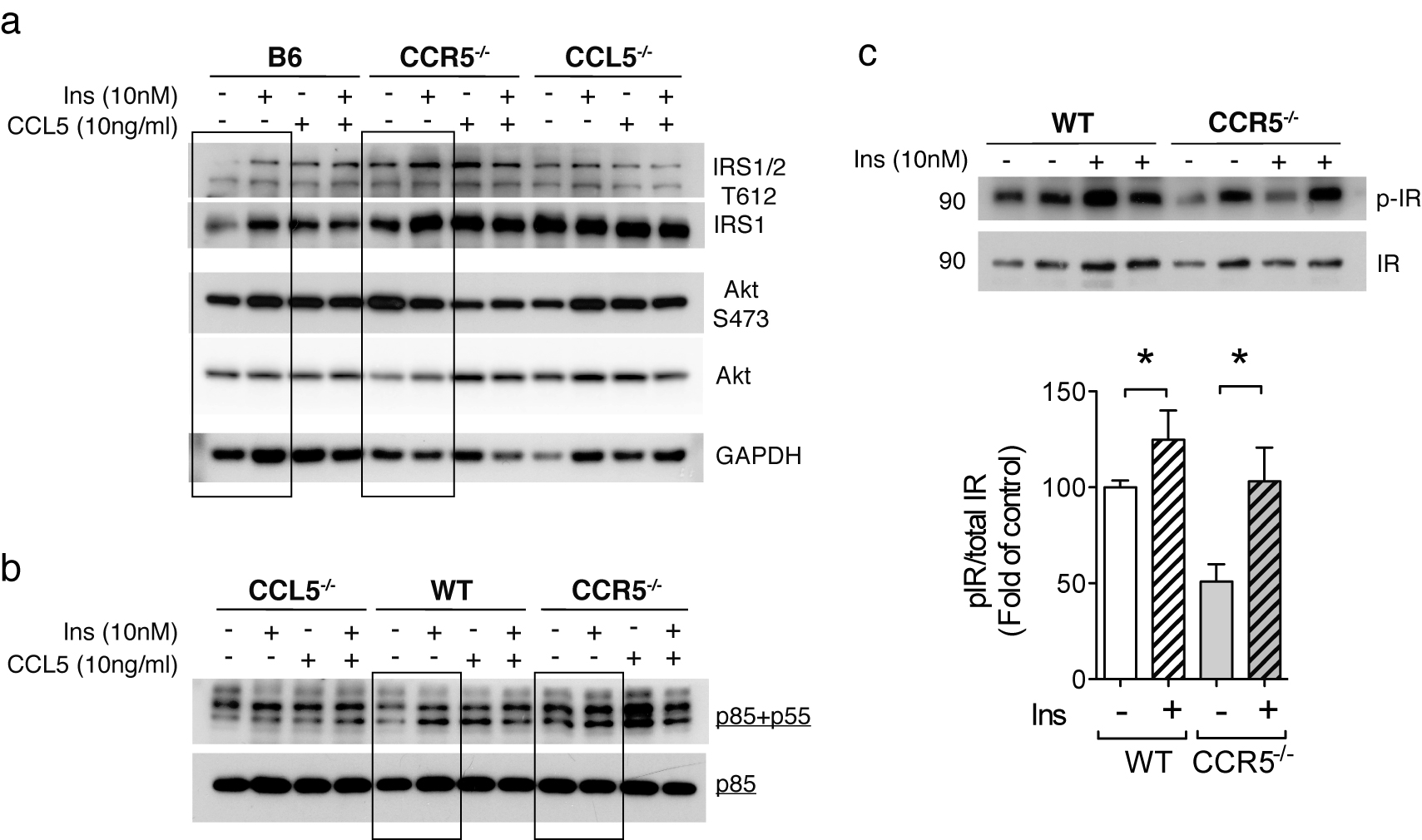


**Supl. Fig. 3: The activation of the insulin-signaling pathway in mouse hypothalamus.** The hypothalamic tissues from WT and CCR5^-/-^ mice were stimulated without or with insulin (10nM) *ex vivo*. (a, b) The activation of insulin down stream signaling molecules as IRS-1, PI3K – p85 and Akt were impaired in CCR5^-/-^ and CCL5^-/-^ hypothalamus as reduced phosphor-IRS1/2 T612, phosphor-p85 and phosphor-AktS473. Boxed bands are also present in Fig. 2a. (c) The activation of the insulin receptor as phosphor-Insulin Receptor (Y1361) in WT and CCR5^-/-^ hypothalamus.


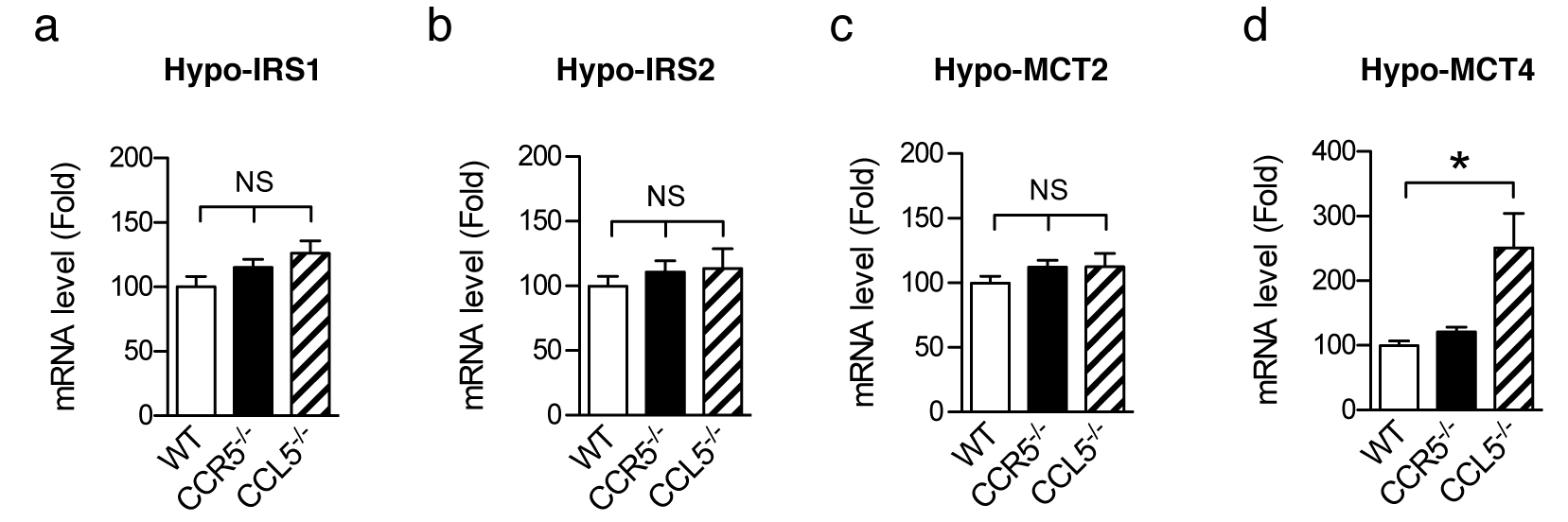


**Supl. Fig. 4: The mRNA levels of IRSs and lactate transporters in WT, CCL5 and CCR5 deficient mouse hypothalamus.** The mRNAs from hypothalamic tissue were extracted from WT mice, CCR5^-/-^ mice and CCL5^-/-^ mice (n=6-8 in each group). The gene expression levels of IRS-1 (**a**), IRS-2 (**b**), and monocarboxylate transporters - MCT-2 (**c**), and MCT-4 (**d**) were analyzed by quantitative PCR. Data were compared to WT in each group after normalization with β-Actin and are presented as mean ± SEM values (*, *p*<0.05; compared to WT groups; NS: no significant difference).


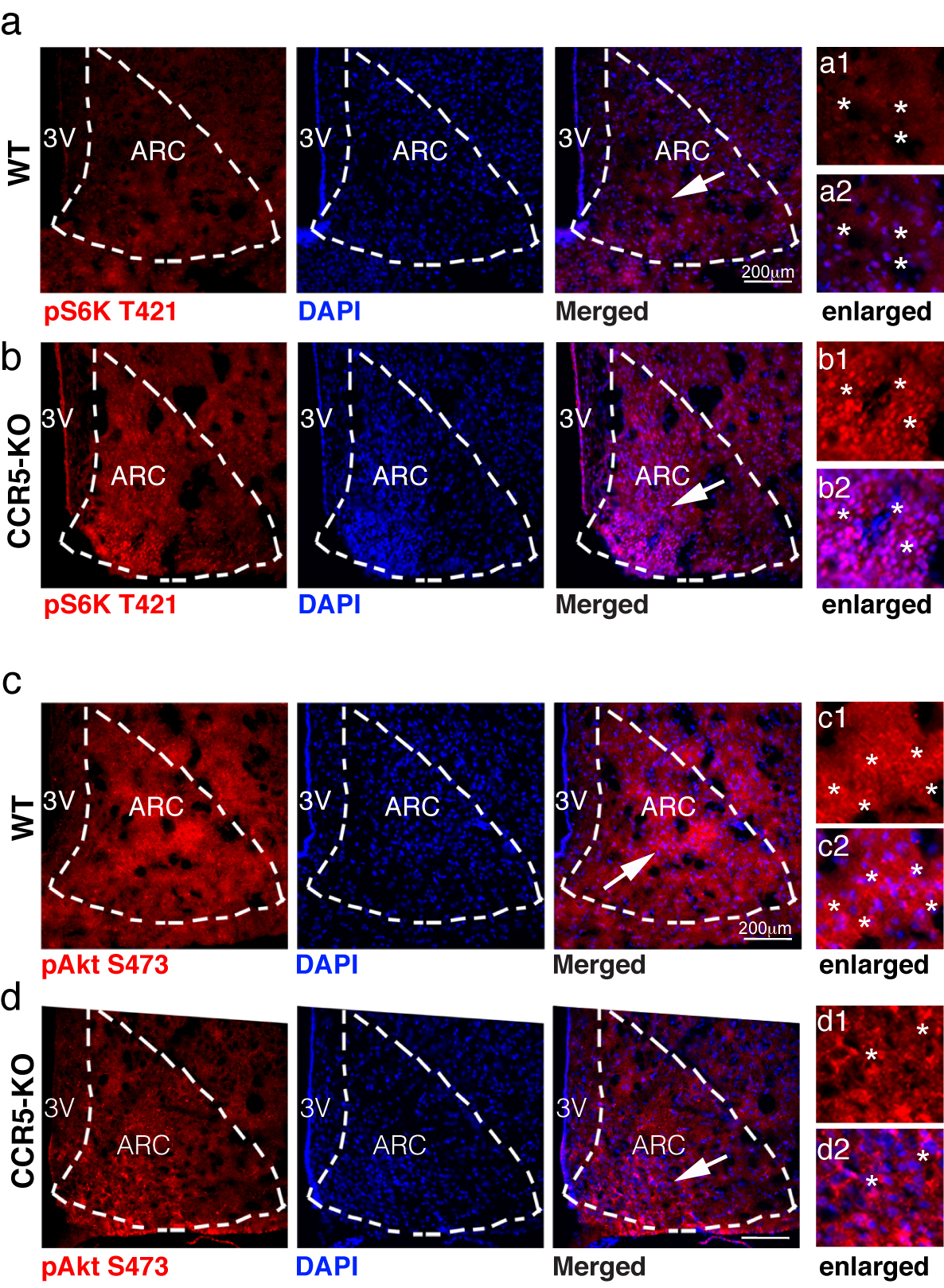


**Supl. Fig. 5: The phosphorylation status of S6 kinase and Akt in mouse hypothalamus.** Mice after feeding were sacrificed and brain tissues were incubated with indicated antibodies. The signal for phosphor-S6K^T421^ in WT ARC was weak (a); large numbers of phosphor-S6K^T421^ positive neurons were found in CCR5^-/-^ ARC region (b). Arrow point regions are enlarged on the right side (a1, a2 for WT; b1, b2 for CCR5^-/-^). Asterisks label the pS6K^T421^ positive neurons. Immunolabeling of phosphor-Akt^S473^ was high in WT ARC (c) but there was a lower phosphor-Akt^S473^ signal in the CCR5^-/-^ (d). Asterisks point to pAkt^S473^ positive neurons in the right panel (c1, c2 for WT; d1, d2 for CCR5^-/-^). DAPI labels the nucleus in blue. The scale bar indicates 200 μm.


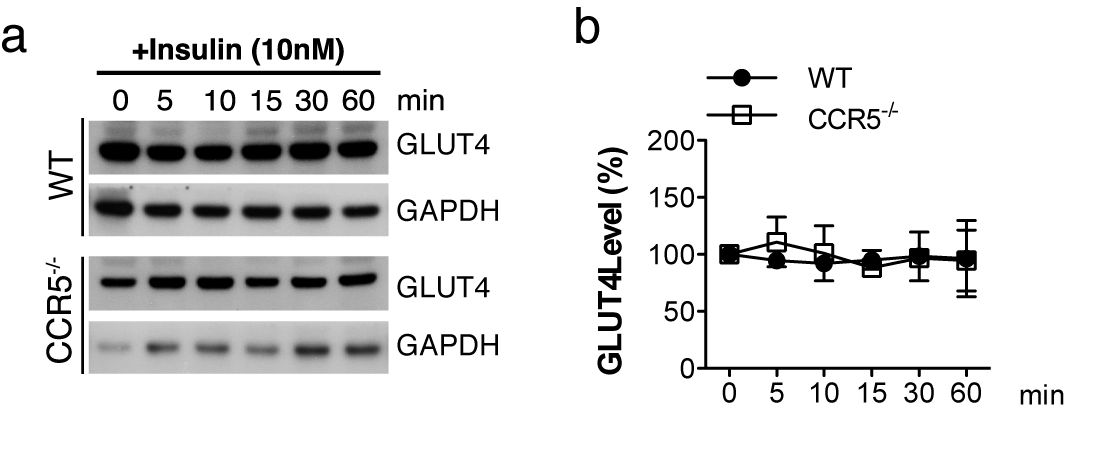


**Supl. Fig. 6: The GLUT4 levels in primary hypothalamic neurons.**

(**a**) The protein levels of cellular GLUT4 in cultured hypothalamus neurons after insulin stimulation. GAPDH was used as protein loading control. (**b**) The quantification of total GLUT4 levels at different time points after insulin stimulation. The level of GLUT4 was not different between each time point in both WT and CCR5^-/-^ cultured hypothalamic neurons.

**
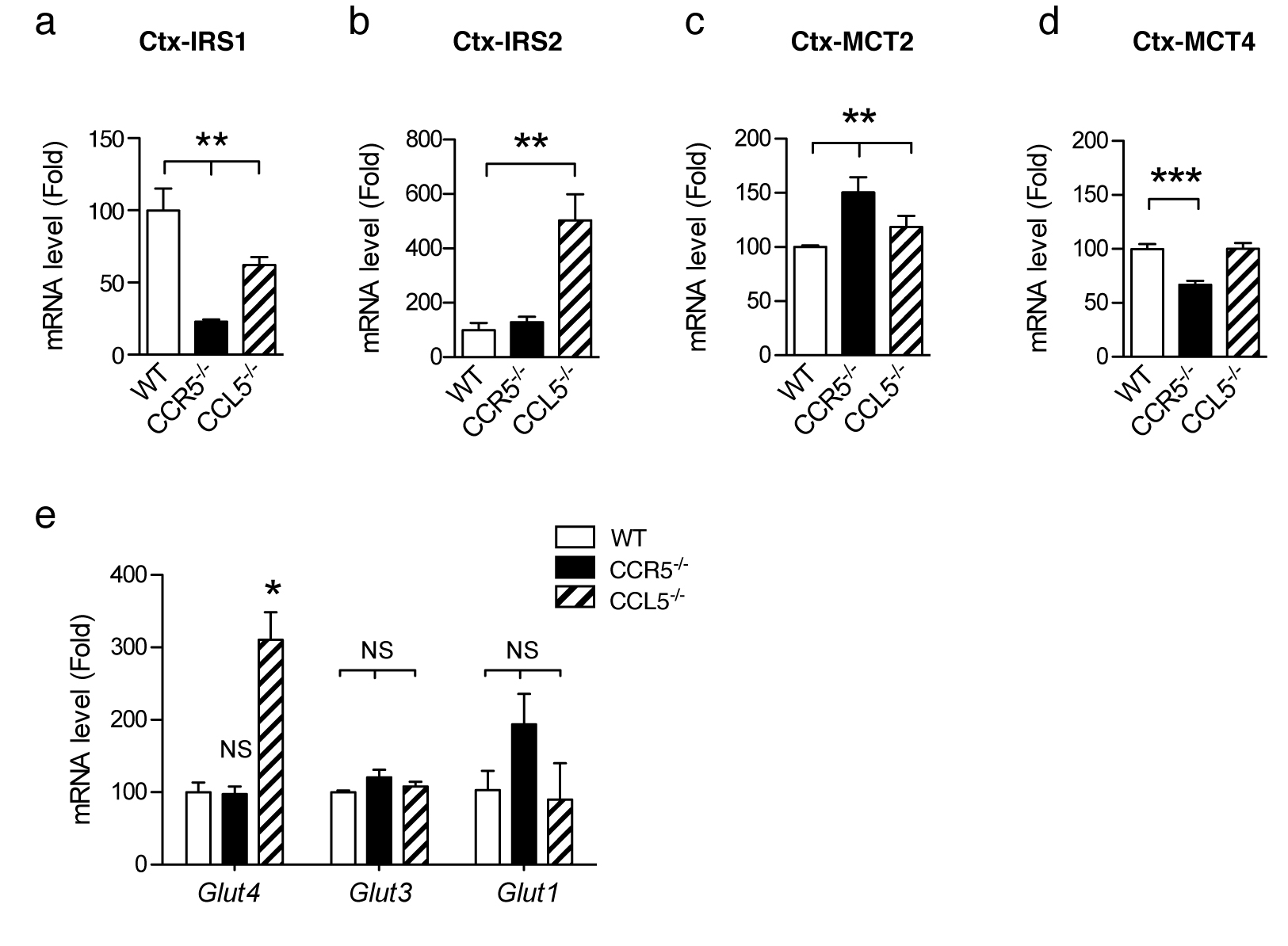
**

**Supl. Fig. 7: The mRNA levels of IRSs, lactate transporters and glucose transporters in CCL5 and CCR5 deficient mouse cortex.** The mRNAs from cerebral cortical tissue were extracted from WT mice, CCR5^-/-^ mice and CCL5^-/-^ mice (n=3~4 in each group). The gene expression levels of IRS-1 (**a**), IRS-2 (**b**), MCT-2 (**c**), and MCT-4 (**d**), were analyzed by quantitative PCR. GLUT4, GLUT3 and GLUT1 mRNA levels showed in (**e**). Data were compared to WT in each group after normalization with β-Actin and are presented as mean ± SEM values (*, *p*<0.05; **, *p*<0.01; ***, *p*<0.001, compared to WT groups. NS: no significant difference).

**
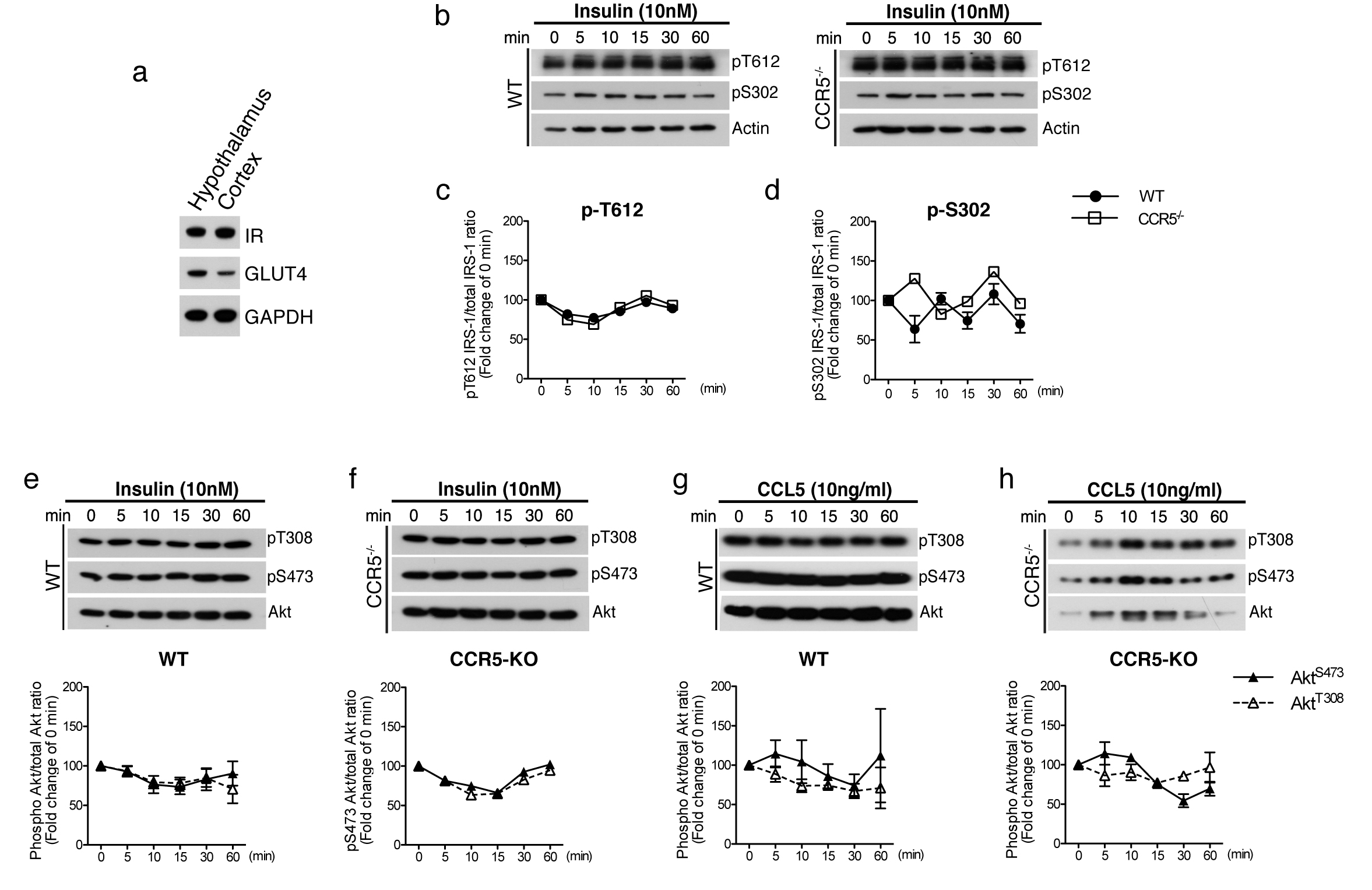
**

**Supl. Fig. 8: The activation of insulin-related signaling pathways in primary cortical neurons.**

(**a**) Protein levels of GLUT4 and insulin receptor in mouse hypothalamus and cortex. (**b**-**f**) Cortical neurons cultured from WT or CCR5^-/-^ mice were stimulated with insulin (10nM) for 0, 5, 10, 15, 30, and 60 min. The insulin signaling pathway proteins, such as insulin response substrate -1 (IRS-1) phosphor - T612 (**b**, **c**) and phosphor - S302 (**b**, **d**), Akt phosphor - S473 and T308 (**e**, **f**) were detected by Western blotting. The activation of Akt S473 and Akt T308 by CCL5 (10ng/ml) in cortical neurons was also analyzed (**g**, **h**).

**
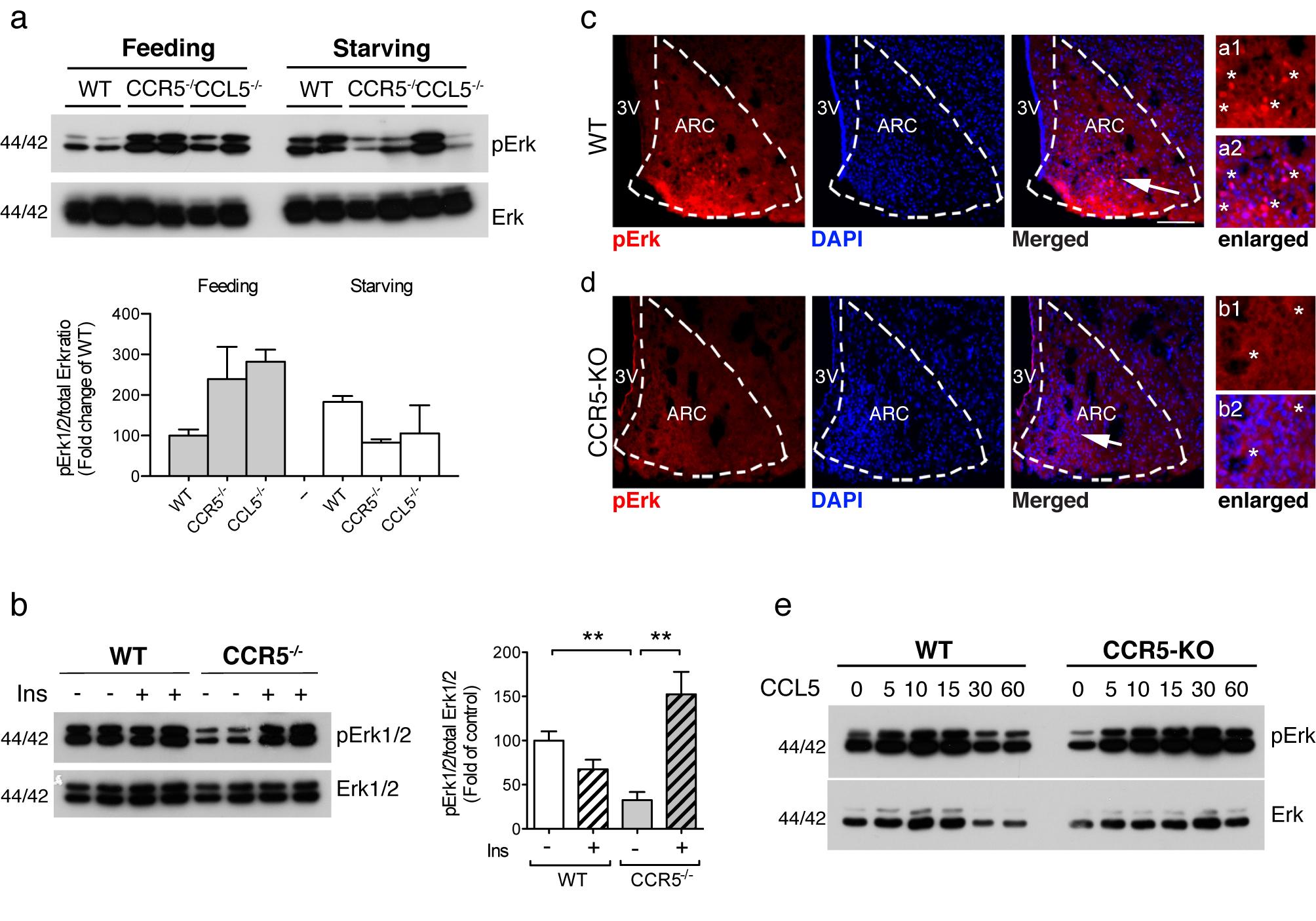
**

**Supl. Fig. 9: Erk1/2 signaling activation by insulin in mice hypothalamus.** (a) Erk1/2 activity in feeding and 8hr fasting mouse hypothalamus. (b) The phosphorylation of Erk1/2 after insulin stimulation *ex vivo*. The immunolabeling of phosphor-Erk1/2 in WT (c) and CCR5^-/-^ (d) ARC neurons. Arrow point regions were enlarged on the right side; Asterisks point to pErk1/2 positive neurons in the right panel (c1, c2 for WT; d1, d2 for CCR5^-/-^). DAPI labeled the nucleus in blue. The scale bar indicates 200 μm. (e) The activation of Erk signaling upon CCL5 in cultured hypothalamic neurons from WT and CCR5-KO mice.

**4. Supplementary Movies:**

**Supplementary Movie 1:** Insulin stimulated GLUT4-GFP movement in WT hypothalamic neurons.

**Supplementary Movie 2:** Insulin stimulated GLUT4-GFP movement in CCR5^-/-^ hypothalamic neurons.

**Supplementary Movie 3:** CCL5/RANTES stimulated GLUT4-GFP movement in WT hypothalamic neurons.

**5. Supplementary Result and Discussion:**

Erk signaling activation upon insulin exposure in hypothalamus was very different from that in peripheral hepatocytes. In hepatocytes, the Erk signaling pathway will be activated by insulin^5^. We found reduced Erk activity in feeding or *ex vivo* insulin stimulated in WT hypothalamic tissue (Supl.Fig. 9a, b) which was increased in CCR5 and CCL5 deficiency (Supl. Fig. 9a, b). In immunostaining assays, some of the WT ARC neurons have very strong immunolabeling of phosphor-Erk1/2, but this was not seen in CCR5^-/-^ (Supl. Fig. 9c, d). We also performed cellular studies to investigate Erk signaling upon CCL5. We found that CCL5 can activate an Erk signal regardless of CCR5 status in hypothalamic neurons (Supl. Fig. 9e). We believe there might be interactions between the insulin receptor and CCL5 signaling with other types of CCL5 receptors such as GPR75 in pancreatic β-cells. GPR75 in pancreatic β-cells can increase insulin secretion and improve glucose homeostasis in mice^6^. Our findings do support that Akt pathway activation of GLUT4 membrane translocation is the major pathway regulated by CCL5-CCR5 in hypothalamus, but additional studies are needed to explore non-CCR5 mediation of CCL5 actions in other tissues.

**6. Supplementary Reference:**

1. Chou, S.Y. *et al.* Expanded-polyglutamine huntingtin protein suppresses the secretion and production of a chemokine (CCL5/RANTES) by astrocytes. *The Journal of neuroscience : the official journal of the Society for Neuroscience* **28**, 3277-3290 (2008).

2. Ju, T.C. *et al.* Nuclear translocation of AMPK-alpha1 potentiates striatal neurodegeneration in Huntington's disease. *The Journal of cell biology* **194**, 209-227 (2011).

3. Lizunov, V.A. *et al.* Insulin stimulates fusion, but not tethering, of GLUT4 vesicles in skeletal muscle of HA-GLUT4-GFP transgenic mice. *American journal of physiology. Endocrinology and metabolism* **302**, E950-960 (2012).

4. Franck, N. *et al.* Insulin-induced GLUT4 translocation to the plasma membrane is blunted in large compared with small primary fat cells isolated from the same individual. *Diabetologia* **50**, 1716-1722 (2007).

5. Zhang, W., Thompson, B.J., Hietakangas, V. & Cohen, S.M. MAPK/ERK signaling regulates insulin sensitivity to control glucose metabolism in Drosophila. *PLoS Genet* **7**, e1002429 (2011).

6. Liu, B. *et al.* The novel chemokine receptor, G-protein-coupled receptor 75, is expressed by islets and is coupled to stimulation of insulin secretion and improved glucose homeostasis. *Diabetologia* **56**, 2467-2476 (2013).
